# Does Ethnicity Matter in Multiple Myeloma Risk Prediction in the Era of Genomics and Novel Agents? Evidence From Real-World Data

**DOI:** 10.3389/fonc.2021.720932

**Published:** 2021-11-09

**Authors:** Akanksha Farswan, Anubha Gupta, Krishnamachari Sriram, Atul Sharma, Lalit Kumar, Ritu Gupta

**Affiliations:** ^1^ Signal Processing and Biomedical Imaging Lab (SBILab), Department of Electronics and Communication, Indraprastha Institute of Information Technology-Delhi, New Delhi, India; ^2^ Department of Computational Biology, Indraprastha Institute of Information Technology-Delhi, New Delhi, India; ^3^ Department of Medical Oncology, Dr. B.R.A. IRCH, AIIMS, New Delhi, India; ^4^ Laboratory Oncology Unit, Dr. Bhim Rao Ambedkar Institute Rotary Cancer Hospital, All India Institute of Medical Sciences (Dr. B.R.A. IRCH, AIIMS), New Delhi, India

**Keywords:** AI in cancer research, ML in cancer survival, risk stratification of multiple myeloma, GMM clustering in cancer, consensus clustering in cancer, hematological malignancy

## Abstract

**Introduction:**

Current risk predictors of multiple myeloma do not integrate ethnicity-specific information. However, the impact of ethnicity on disease biology cannot be overlooked. In this study, we have investigated the impact of ethnicity in multiple myeloma risk prediction. In addition, an efficient and robust artificial intelligence (AI)-enabled risk-stratification system is developed for newly diagnosed multiple myeloma (NDMM) patients that utilizes ethnicity-specific cutoffs of key prognostic parameters.

**Methods:**

K-adaptive partitioning is used to propose new cutoffs of parameters for two different datasets—the MMIn (MM Indian dataset) dataset and the MMRF (Multiple Myeloma Research Foundation) dataset belonging to two different ethnicities. The Consensus-based Risk-Stratification System (CRSS) is designed using the Gaussian mixture model (GMM) and agglomerative clustering. CRSS is validated *via* Cox hazard proportional methods, Kaplan–Meier analysis, and log-rank tests on progression-free survival (PFS) and overall survival (OS). SHAP (SHapley Additive exPlanations) is utilized to establish the biological relevance of the risk prediction by CRSS.

**Results:**

There is a significant variation in the key prognostic parameters of the two datasets belonging to two different ethnicities. CRSS demonstrates superior performance as compared with the R-ISS in terms of C-index and hazard ratios on both the MMIn and MMRF datasets. An online calculator has been built that can predict the risk stage of a multiple myeloma (MM) patient based on the values of parameters and ethnicity.

**Conclusion:**

Our methodology discovers changes in the cutoffs with ethnicities from the established cutoffs of prognostic features. The best predictor model for both cohorts was obtained with the new ethnicity-specific cutoffs of clinical parameters. Our study also revealed the efficacy of AI in building a deployable risk prediction system for MM. In the future, it is suggested to use the CRSS risk calculator on a large dataset as the cohort size of the present study is 25% of the cohort used in the R-ISS reported in 2015.

## Introduction

Multiple myeloma is a hematopoietic malignancy of plasma cells with an overall survival period ranging from 6 months to more than 10 years. The variability in the outcome of patients is an implication of the clinical and biological heterogeneity underlying multiple myeloma (MM). Substantial advances in tumor biology have made it possible to dissect the tumor heterogeneity present in MM, optimize patient treatment, and examine patient outcome. Multiple prognostic systems (1–5) have been described in MM that stratify patients into different risk groups. These risk groups further assist in identifying high-risk patients who may require intense therapy upfront and/or a higher monitoring frequency during the follow-up periods. The first staging system for MM was proposed in 1975 (1) followed by the development of the International Staging System (ISS) (2) in 2005 and a Revised ISS (R-ISS) (3) in 2015. The ISS utilizes serum albumin and beta2-microglobulin, while the R-ISS makes use of ISS, lactate dehydrogenase (LDH), and high-risk cytogenetic aberrations (HRCA). Currently, triplet combination therapy is the new standard of care in MM which has shifted many high-risk patients to standard-risk category, thereby justifying the need for a new risk-stratification system with the possibility of inclusion of more prognostic factors.

Although human physiological and genetic profile is known to vary across ethnic groups, the current MM risk-staging systems do not account for ethnicity-specific information that can have a huge impact on the risk score prediction. It is evident from the studies that African Americans experience two to three times higher incidence rates than Asians, Mexican-Americans, or Europeans (6). Recent studies have observed a significant variation in the overall survival of different groups belonging to distinct races/ethnicities since the introduction of novel treatment agents in MM (7–10). In a recent study, vitamin D deficiency at diagnosis was found to be a predictor of poor overall survival in MM (11). However, this was significant only for White Americans and not for African Americans even at lower cutoffs of deficiency (11). Similarly, HRCA, which is used to determine the intensity of frontline therapy, does not track with survival outcomes in African Americans (10), thereby highlighting the need for a race-specific risk-stratification system. Though ethnicity is an important prognostic factor in predicting the risk for MM (12), the variations in the clinical characteristics among the different ethnic groups have not been evaluated adequately. Therefore, it is desirable to have a staging system that includes the variations in the clinical characteristics of the patients pertaining to distinct ethnic groups. In addition, it should be based on clinical and laboratory parameters that are easily accessible in healthcare settings across the globe. Therefore, to address this concern, we first investigated the role of ethnicity in the differential clinical characteristics in the two independent cohorts of MMIn and MMRF patients with newly diagnosed multiple myeloma (NDMM) belonging to two separate ethnic groups. Furthermore, we proposed the Consensus based Risk-Stratification System (CRSS), an AI-enabled risk-stratification system, for NDMM that incorporates the ethnicity-specific cutoffs of the laboratory parameters like albumin, beta-2 microglobulin (β2M), calcium, estimated glomerular filtration rate (eGFR), hemoglobin, and age along with HRCA. The newly proposed ethnicity-aware AI-assisted CRSS method was shown to have superior performance as compared with R-ISS. In addition, we also interpreted our proposed model *via* SHapley Additive exPlanations (SHAP) (13) analysis to demonstrate the clinical significance of the risk stage predictions by CRSS. Our findings establish the significance of integrating ethnicity-specific information as well as the effectiveness of machine learning methods in devising a robust risk-staging model for MM.

## Materials and Methods

### Datasets

A total of 1,675 entries were found in the computerized database search on June 28, 2019, with the keyword “ICD C90” registered at the Institute Rotary Cancer Centre, All India Institute of Medical Sciences (AIIMS). Patients with plasma cell dyscrasia other than MM (*n* = 253) or who were lost to follow-up after a single visit (*n* = 111) or before first response could be assessed (*n* = 21) or with inadequate clinical and/or laboratory parameters (*n* = 121) or with early deaths (*n* = 99) were excluded. The remaining 1,070 patients of MM belonging to the Indian population, referred to as MMIn, were evaluated in this study (**Figure S1**). Out of 1,070 patients, 41 patients had one or two missing values. There are several methods to impute missing values (14–17). However, in the MMIn dataset, missing values were imputed with the median value of the parameters. An independent cohort of 900 MM patients enrolled in the Multiple Myeloma Research Foundation (MMRF) repository was also used for developing the model. Clinical and laboratory data for the MMRF dataset, belonging to the American population, are available publicly. High-risk cytogenetic information was available for 384 out of 1,070 patients in the MMIn cohort and 800 out of a total of 900 patients in the MMRF which were further used for building the staging model.

### Clinical and Laboratory Characteristics

The clinical, laboratory, and radiological data were obtained from the medical case files. The R-ISS could be assigned to a subset of patients (*n* = 627) as described previously (18). Response outcome was estimated following the international uniform response criteria for multiple myeloma (19). Progression-free survival (PFS) was computed from the date of diagnosis till the time of progression or death. Overall survival (OS) was computed from the date of diagnosis till death due to any cause or being censored at last follow-up. Baseline clinical and laboratory features of the patients are given in **Supplementary Table S1**.

### Study Design

**Figure 1:**
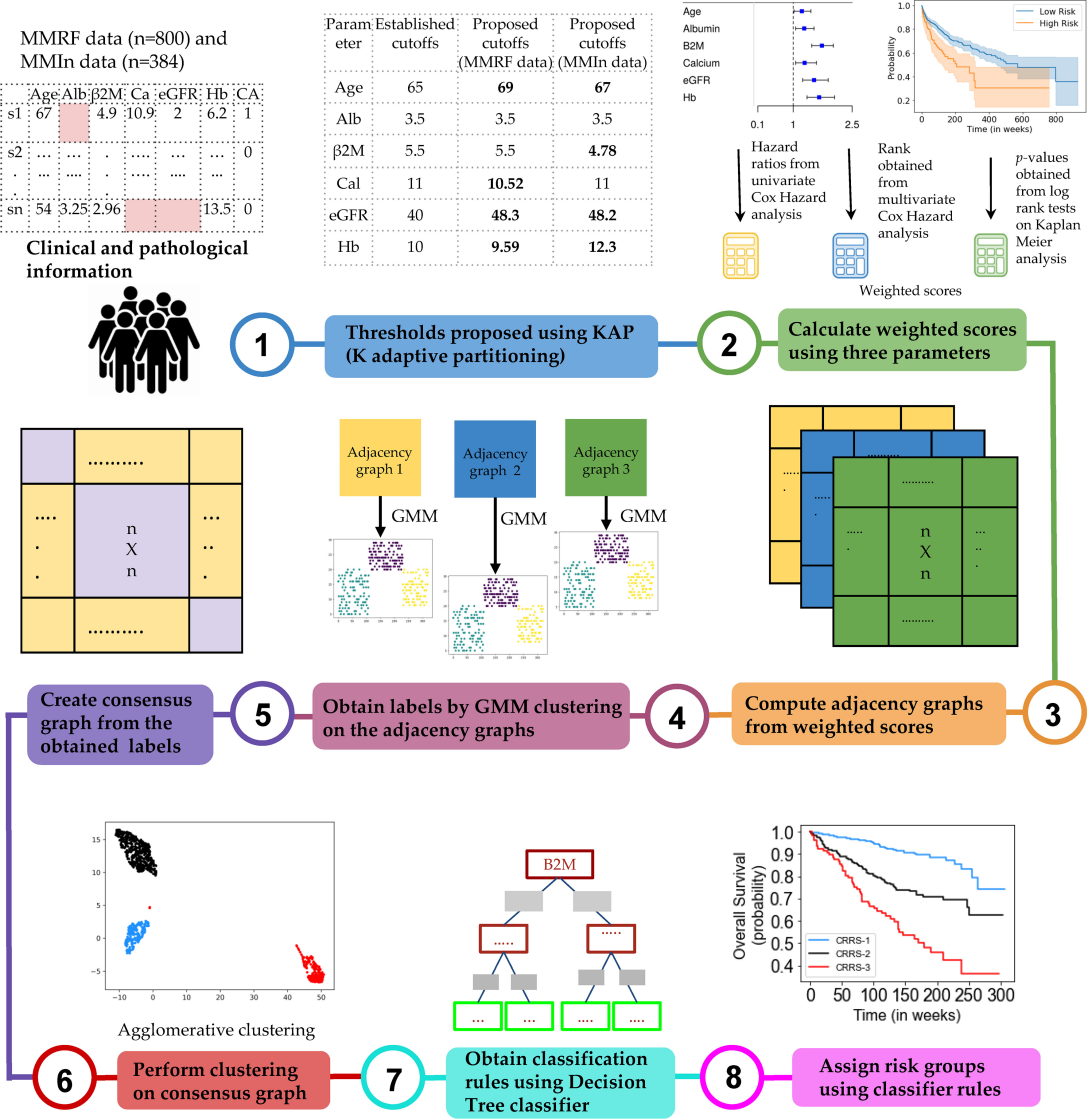
Workflow for the development of the Consensus-based Risk-Stratification System (CRSS) for newly diagnosed multiple myeloma patients.

The complete design strategy of the consensus-based approach for developing the risk-stratification system (CRSS) is explained in this section (**Figure 1**). Data from both cohorts were separately used to develop the risk-staging models based on CRSS. Different clinical parameters were evaluated for developing the risk-staging system consisting of age, albumin, β2M, calcium, eGFR, hemoglobin, LDH, and HRCA which includes t(4;14), t(14;16), and del17. β2M and LDH levels are reflective of tumor burden and serum albumin, hemoglobin, calcium, and creatinine are reflective of the bone and renal homeostasis. eGFR was calculated from creatinine concentration using the MDRD eGFR equation (20). LDH values were brought to a common scale by multiplying each entry by 280 and dividing it by the upper limit of LDH provided for that particular entry in MMIn data. Description of the steps used in the consensus-based approach for developing the risk-staging model is given below:

*Step 1: Dividing patients into two risk groups based on established thresholds of parameters*. For each parameter, patients were initially divided into high-risk and low-risk groups using the well-established cutoffs of these parameters (21) as shown in **Table 1**. Established thresholds for albumin and β2M are derived from the ISS, and for eGFR, calcium, and hemoglobin, the thresholds are derived from the revised IMWG criteria (21).

**Table 1:** Comparison of established and proposed cutoffs for clinical and laboratory parameters for the stratification of patients for progression-free survival (PFS) and overall survival (OS) in MMIn and MMRF using Kaplan–Meier analysis.

Parameter	Established cutoff value	Proposed cutoff value	PFS		OS	
			*p*-value with established cutoff	*p*-value with proposed cutoff	*p*-value with established cutoff	*p*-value with proposed cutoff
**MMIn (*n* = 1,070)**						
Age (years)	>65	**>67**	0.11	**0.012**	5.84e-5	**1.25e-6**
Albumin (g/dl)	≤3.5	≤3.5	0.115	0.115	7.0e-4	7.0e-4
β2M (mg/L)	≥5.5	**≥4.78**	**8.15e-10**	9.32e-10	4.13e-10	**4.53e-14**
Calcium (mg/dl)	≥11	≥11	0.0078	0.0078	0.0037	0.0037
eGFR (ml/min/1.73m^2^)	≤40	**≤48.2**	0.16	**0.04**	0.005	**1.5e-4**
Hb (g/dl)	≤10	**≤12.3**	0.0019	**8.56e-5**	0.0014	**3.75e-7**
**MMRF (*n* = 900)**						
Age (years)	>65	**>69**	3.23e-05	**1.98e-08**	1.06e-05	**1.58e-09**
Albumin (g/dl)	≤3.5	≤3.5	0.00017	0.00017	8.47e-07	8.47e-07
β2M (mg/L)	≥5.5	≥5.5	1.22e-10	1.22e-10	9.25e-13	9.25e-13
Calcium (mg/dl)	≥11	**≥10.52**	0.0077	**1.40e-04**	5.88e-06	**3.49e-06**
eGFR (ml/min/1.73m^2^)	≤40	**≤48.3**	4.5e-05	**4.67e-09**	7.48e-06	**2.48e-10**
Hb (g/dl)	≤10	**≤9.59**	2.82e-06	**5.69e-09**	6.77e-06	**5.42e-07**

The proposed cutoffs were found using complete data of MMIn (n = 1,070) and MMRF (n = 900). Less than or equal to cutoff reveals the increased risk in the patient. “>65” shows that a patient with age greater than 65 years is at greater risk than a patient less than 65 years. “≤3.5” shows that a patient with albumin levels less than equal to 3.5 is at a greater risk than a patient with albumin levels greater than 3.5. It holds true for other parameters also in a similar manner. Bold values of the column “proposed cutoff value” signify the change in the value of the parameters from the existing cut-offs. p-values in bold signify that p-values became more significant with the proposed changes in cutoffs.

*Step 2: Finding new thresholds of parameters via KAP*. The K-adaptive partitioning (22) (KAP) algorithm was used to find new threshold values for the parameters using complete data of MMIn (*n* = 1,070) and MMRF (*n* = 900). KAP was performed on the parameters of the patients yielding two threshold values for each parameter, one from PFS and the other from OS analysis. The cutoff which was close to the original value was chosen as the new cutoff for each parameter. Patients were again divided into high- and low-risk groups based on the proposed cutoffs. The proposed thresholds maximized the separation between high- and low-risk groups as compared with the established thresholds. This is evident from the lower *p*-values obtained from the log-rank test on the Kaplan–Meier curves for all the parameters. A complete list of the proposed thresholds for the MMIn and MMRF data is shown in **Table 1**.

*Step 3: Cumulative integration of the prognostic impact of the parameters*. The collective prognostic impact of the parameters was integrated into risk staging *via* creation of three different adjacency graphs using hazard ratios obtained from univariate Cox hazard analysis, *p*-values obtained from log-rank test on Kaplan–Meier curves, and ranks obtained from multivariate Cox hazard analysis.

*Step 4: Creation of the first adjacency graph*. The first adjacency graph was created using ranks obtained from the multivariate Cox hazard analysis. The parameter with the highest hazard value was given the highest rank, and the one with the lowest hazard value was given the lowest rank. The respective ranks served as the weights of each of the parameters and captured the relative impact of each parameter on the survival of patients. Next, the risk score for each patient was calculated by successive addition of the weights of all those parameters that had values (in the respective patient) greater than the cutoffs defined for the high-risk group. These patient scores were used to compute an adjacency graph of *n* rows and *n* columns (columns are features), where *n* is the number of patients. Each row corresponds to one patient and each entry in the row is the absolute difference between the score of that patient with each of the patients including self.

*Step 5: Creation of the second and third adjacency graphs*. For the second adjacency graph, hazard ratio values obtained from univariate Cox hazard analysis were used. For each parameter, the highest of the two HR values obtained from PFS and OS was chosen and normalized using “minmax” scaling. The scaled HR values were assigned as the respective weights of each of the parameters representing the impact of each parameter on the survival of patients. The third adjacency graph was created using *p*-values obtained by performing a log-rank test on Kaplan–Meier curves. For each parameter, the lower of the two *p*-values obtained from PFS and OS was chosen and normalized using “minmax” scaling. The scaled *p-*values were assigned as the respective weights of each of the parameters. Furthermore, the risk score for each patient was calculated by successive addition of the weights of all those parameters that had values (in the respective patient) greater than the cutoff defined for the high-risk group. The two different patient scores obtained from univariate hazard ratios and *p*-values were further used to compute two separate adjacency graphs of *n* rows and *n* columns (columns are features), where *n* is the number of patients. Each row corresponds to one patient and each entry in the row is the absolute difference between the score of that patient with each of the patients including self.

*Step 6: Gaussian mixture model (GMM) clustering on the adjacency graphs*. GMM-based clustering is an unsupervised clustering algorithm which was applied on the three adjacency graphs to obtain clustering labels.

*Step 7: Creation of a consensus graph*. The clustering outputs of the three different adjacency graphs were used to create a consensus graph (23) of size *n* × *n*. The entry for the *i*th row and *j*th column in the consensus graph was determined by calculating the number of times *i*th and *j*th patients were assigned the same group. Diagonal entries were zero in this graph.

*Step 8: Hierarchical clustering on the consensus graph*. Agglomerative clustering was performed on the consensus graph to cluster the patients into three risk groups. Each cluster of patients was assigned one label: stage 1 (low risk), stage 2 (intermediate risk), or stage 3 (high risk). The rationale behind using multiple clustering was to combine the results of the clustering outputs achieved from the different adjacency graphs and ensure the stability of the final clusters deduced from agglomerative clustering.

*Step 9: Training a decision tree classifier*. The staging labels obtained from agglomerative clustering served as ground-truth labels for training the supervised decision tree classifier. The trained decision tree classifier provided the rules in terms of the parameters for the identification of risk groups, labeled as CRSS-1 (low risk), CRSS-2 (intermediate risk), and CRSS-3 (high risk) (**Figures S2, S3**).

*Step 10: Infer actual risk groups of the patients using decision tree classifier rules.* Decision tree classifier rules were then used to identify the risk stages of the patients in both cohorts. The risk stage assigned by the decision tree classifier was considered the actual risk class for each patient.

### Creation of Multiple Models on the Datasets

The CRSS method explained in **Figure 1** was used to create multiple models for the MMIn and MMRF datasets. Models A1, A2, and A3 were built for the MMIn data. Model A1 was built using established cutoffs of the parameters of albumin, β2M, LDH, and HRCA. Model A2 was built using the established cutoffs of the parameters of albumin, age, calcium, eGFR, hemoglobin, β2M, and HRCA. Model A3 uses the same parameters as model A2, but with the newly proposed cutoffs of the parameters derived from the MMIn dataset. Similarly, models M1, M2, M3, and M4 were built for the MMRF data. Models M1 and M2 are equivalent to models A1 and A2, respectively. For model M3, the proposed cutoffs of parameters derived from the MMIn dataset were used for albumin, age, calcium, eGFR, hemoglobin, β2M, and HRCA. Model M4 is similar to model M3, but uses the proposed cutoffs of the parameters derived from the MMRF dataset.

## Results

### Clinical and Laboratory Characteristics of Myeloma Patients

The baseline clinical and laboratory features of patients from the two cohorts were compared using unpaired Wilcoxon rank-sum test. The median values of all the parameters except albumin were found to be significantly different (*p*-value < 0.05, **Table S2**) in both cohorts thereby substantiating that the two populations are different. Novel agents (IMIDs: thalidomide or lenalidomide and/or PSI, i.e., bortezomib) either as primary or maintenance therapy were given to all the patients. Triplet therapy was rendered to 56.5% of the patients. With a median follow-up of 166 weeks (range: 14–961 weeks), 626 patients progressed (median PFS = 117 weeks) and 372 died (median OS = 166 weeks).

### Results on the MMIn Dataset (*n* = 384)

**Figure 2:**
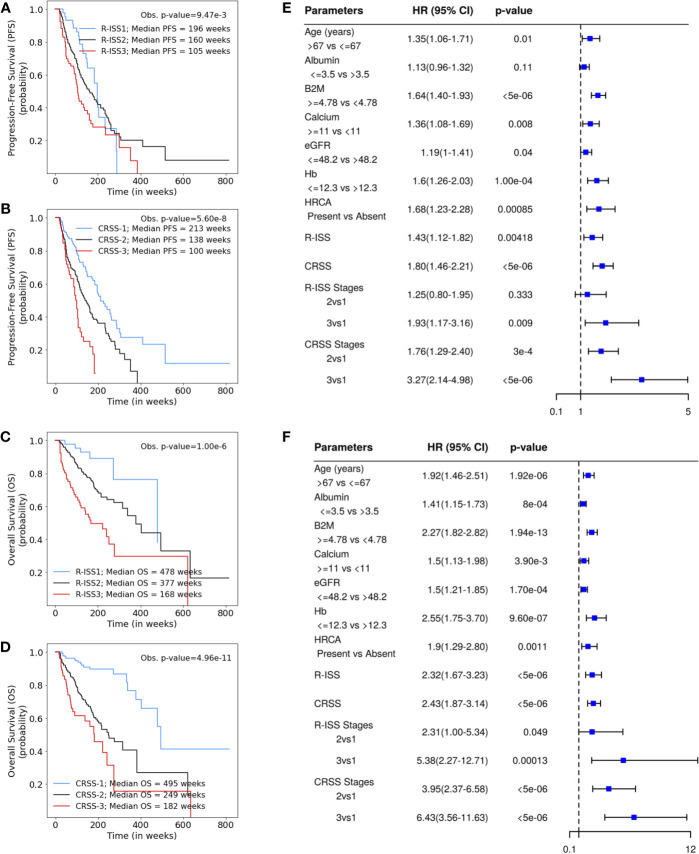
**(A, B)** Progression-free survival in patients with multiple myeloma (MM) from the MMIn cohort (*n* = 1,070) stratified by the Revised International Staging System (R-ISS) (*n* = 355) and the proposed CRSS (*n* = 384), respectively. R-ISS1 is the low-risk stage, R-ISS2 is the intermediate-risk stage, and R-ISS3 is the high-risk stage. Median progression-free survival (PFS) for R-ISS1, R-ISS2, and R-ISS3 are 196, 160, and 105 weeks, respectively. The observed *p*-value obtained after performing a log-rank test on R-ISS is 9.47e-3. Similarly, CRSS-1 is the low-risk stage, CRSS-2 is the intermediate-risk stage, and CRSS-3 is the high-risk stage. Median PFS for CRSS-1, CRSS-2, and CRSS-3 are 213, 138, and 100 weeks, respectively. The observed *p*-value obtained after performing a log-rank test on CRSS is 5.60e-8. **(C, D)** Overall survival in patients with MM from the MMIn cohort (*n* = 1,070) stratified by the R-ISS (*n* = 355) and CRSS (*n* = 384), respectively. Median overall survival (OS) for R-ISS1, R-ISS2, and R-ISS3 are 478, 337, and 168 weeks, respectively. The observed *p*-value obtained after performing a log-rank test on R-ISS is 1.00e-6. Median OS for CRSS-1, CRSS-2, and CRSS-3 are 495, 249, and 182 weeks, respectively. The observed *p*-value obtained after performing a log-rank test on CRSS is 4.96e-11. **(E, F)** Univariate Cox hazard analysis on the prognostic factors—age, albumin, beta-2 microglobulin (β2M), calcium, estimated glomerular filtration rate (eGFR), hemoglobin, and high-risk cytogenetic abnormalities (HRCA)—for PFS and OS, respectively. Hazard ratios for all the parameters except HRCA were calculated on complete data (*n* = 1,070) for the MMIn dataset. Hazard ratio for HRCA and the risk-staging models were found using the data for which HRCA information was present (*n* = 384 for the MMIn dataset).

Univariate Cox analysis of the entire patient cohort (*n* = 1,070, **Table S3**, **Figure 2**) revealed increased risk of progression and mortality for age >67 years, albumin ≤3.5, β2M ≥4.78, calcium ≥11, eGFR ≤48.2, and hemoglobin ≤12.3. Multivariate Cox hazard analysis was also performed to analyze the cumulative risk of the parameters (**Table S4**). Of the three models generated, model A3 based on ML-derived cutoffs for the prognostic parameters was the best with higher C-index and hazard ratio (**Table 2**). Using model A3, the patients were risk stratified and the largest proportion of patients were placed in CRSS-2 (*n* = 192, 50%) followed by CRSS-1 (*n* = 137, 35.68%) and CRSS-3 (*n* = 55, 14.32%). KM survival analysis of CRSS groups indicated statistically significant difference in PFS between CRSS-1 and CRSS-2 groups (median PFS: 213 vs. 138 weeks; *p* = 0.0003) and between CRSS-2 and CRSS-3 groups (median PFS: 138 vs. 100 weeks; *p* = 0.0026) (**Figure 2**). For R-ISS, there was a statistically significant difference in PFS between R-ISS2 and R-ISS3 (median PFS: 160 vs. 105 weeks; *p* = 0.01) but not between R-ISS1 and R-ISS2 (median PFS = 196 vs. 160 weeks; *p* = 0.31). Furthermore, for CRSS, there was statistically significant difference in OS between CRSS-1 and CRSS-2 groups (median OS = 495 vs. 249 weeks; *p* = 1.08e-8) as well as between CRSS-2 and CRSS-3 groups (median OS = 249 vs. 182 weeks; *p* = 0.02). For R-ISS, there was statistical difference in OS between R-ISS2 and R-ISS3 groups (median OS = 377 vs. 168 weeks; *p* = 1.86e-5) as well as between R-ISS1 and R-ISS2 groups (median OS = 478 vs. 377 weeks; *p* = 0.03).

**Table 2:** Comparison of different models devised for the risk stratification of patients in the MMIn and MMRF cohorts with the R-ISS.

		PFS			OS		
		Hazard ratio	*p*-value	C-index	Hazard ratio	*p*-value	C-index
**MMIn (*n* = 384)**							
R-ISS (*n* = 355)		1.42	0.004	0.57	2.32	<5e-6	0.636
	2vs1	1.24	0.33		2.31	0.04	
	3vs1	1.92	0.009		5.37	0.00013	
Model A1		1.5	1.00e-5	0.594	2.03	<5e-6	0.646
	2vs1	1.53	0.007		2.13	0.0013	
	3vs1	2.26	2.00e-5		4.16	<5e-6	
Model A2		1.4	0.0001	0.579	1.74	1.00e-5	0.616
	2vs1	1.42	0.056		1.9	0.02	
	3vs1	1.98	0.00013		3.13	2.00e-5	
Model A3 (CRSS)		**1.8**	<5e-6	**0.6**	**2.43**	<5e-6	**0.67**
	2vs1	**1.76**	3.00e-4		**3.95**	<5e-6	
	3vs1	**3.27**	<5e-6		**6.43**	<5e-6
**MMRF (*n* = 800)**							
R-ISS (*n* = 658)		1.61	0.00001	0.578	2.26	<5e-6	0.618
	2vs1	1.49	0.015		1.79	0.03	
	3vs1	2.6	0.00001		4.66	<5e-6	
Model M1		1.55	<5e-6	0.6	2.07	<5e-6	0.656
	2vs1	1.55	0.00042		2.06	0.00067	
	3vs1	2.4	<5e-6		4.3	<5e-6	
Model M2		1.62	<5e-6	0.6	2.36	<5e-6	0.657
	2vs1	1.44	0.01		2.12	0.0081	
	3vs1	2.54	<5e-6		5.22	<5e-6	
Model M3		1.54	<5e-6	0.604	2.2	<5e-6	0.679
	2vs1	1.87	<5e-6		2.95	<5e-6	
	3vs1	2.32	<5e-6		5.11	<5e-6	
Model M4 (CRSS)		**1.79**	<5e-6	**0.61**	**2.85**	<5e-6	**0.676**
	2vs1	**1.76**	8.10e-4		**4.1**	3.40e-4	
	3vs1	**3.19**	<5e-6		**10.61**	<5e-6	

Models were built using data for which high-risk cytogenetic information (HRCA) was available (n = 384 for MMIn and n = 800 for MMRF). R-ISS information was available for only 355 out of 384 patients in the MMIn dataset and 658 out of 800 patients in the MMRF dataset. The model with the best performance was A3 and M4 (in bold).

Model A1: beta-2 microglobulin (β2M), albumin, LDH, and CA [del17, t(4;14), t(14;16)] at existing cutoffs. Model A2: age, β2M, albumin, calcium, estimated glomerular filtration rate (eGFR), Hb, and HRCA using existing cutoffs. Model A3: age, β2M, albumin, calcium, eGFR, Hb, and HRCA using proposed cutoffs for MMIn data. Model M1: β2M, albumin, LDH, and HRCA at existing cutoffs. Model M2: age, β2M, albumin, calcium, eGFR, Hb, and HRCA using existing cutoffs. Model M3: age, β2M, albumin, calcium, eGFR, Hb, and HRCA using proposed cutoffs for MMIn data. Model M4: age, β2M, albumin, calcium, eGFR, Hb, and HRCA using proposed cutoffs for MMRF data.

C-statistic and hazard ratios computed on CRSS surpassed the C-index and hazard ratios obtained for R-ISS with respect to both PFS and OS (**Table 2**). C-statistic for CRSS was 0.60 [Akaike information criteria (AIC) = 2,171.49, Bayesian information criteria (BIC) = 2,175.43, HR = 1.80, 95% CI = 1.46–2.21, *p* < 5e-6] for PFS and 0.67 (AIC = 1,244.72, BIC = 1,248.67, HR = 2.43, 95% CI = 1.87–3.14, *p* < 5e-6) for OS, while C-statistic for R-ISS was 0.57 (AIC = 2,011.14, BIC = 2,015.01, HR = 1.43, 95% CI = 1.12–1.82, *p* = 4.18e-3) for PFS and 0.636 (AIC = 1,132.20, BIC = 1,136.07, HR = 2.32, 95% CI = 1.67–3.23, *p* < 5e-6) for OS.

### Results on the MMRF Dataset (*n* = 800)

For the MMRF data, out of the four models generated, model M4 performed the best and had the highest C-index and hazard ratios as compared with the other models as well as R-ISS (**Table 2**). In the univariate Cox hazard analysis of the MMRF data, risk of progression and mortality was increased for age >69 years, β2M ≥5.5, albumin ≤3.5, hemoglobin ≤9.59, eGFR ≤48.3, and calcium ≥10.52 (**Table S3**, **Figure S4**). Multivariate Cox hazard analysis was also performed (**Table S4**). In the MMRF cohort, using the M4 model, the majority of the patients were placed in CRSS-2 (*n* = 452, 56.5%) followed by CRSS-3 (*n* = 174, 21.75%) and CRSS-1 (*n* = 174, 21.75%). Results of the median PFS on CRSS groups (*p* = 8.64e-12) and R-ISS groups (*p* = 1.73e-5) as well as median OS on CRSS groups (*p* = 1.08e-15) and R-ISS groups (*p* = 6.57e-8) reveal the superior performance of the CRSS than the R-ISS (significant *p*-values; **Figure S4**).

C-statistic for CRSS in MMRF data is 0.61 (AIC = 4,126.07, BIC = 4,130.74, HR = 1.79, 95% CI = 1.52–2.12, *p* < 5e-6) for PFS and 0.676 (AIC = 1,819.95, BIC = 1,824.62, HR = 2.85, 95% CI = 2.19–3.71, *p* < 5e-6) for OS. C-statistic for R-ISS is 0.578 (AIC = 3,413.36, BIC = 3,416.49, HR = 1.61, 95% CI = 1.30–2.00, *p* = 1.00e-5) for PFS and 0.618 (AIC = 1,586.78, BIC = 1,591.27, HR = 2.26, 95% CI = 1.65–3.11, *p* < 5e-6) for OS (**Table 3**).

**Table 3:** Prediction of progression-free survival and overall survival (in %) for CRSS and R-ISS at 1, 2, 3, 4, and 5 years in the MMIn (*n* = 384) and MMRF datasets (*n* = 800).

		MMIn data					
		R-ISS (*n* = 355)			CRSS (*n* = 384)		
	Year	1	2	3	1	2	3
PFS	1	0.9318	0.8305	0.6967	0.8966	0.7812	0.7196
	2	0.8606	0.6601	0.5223	0.7709	0.6265	0.4472
	3	0.6404	0.5124	0.3632	0.6449	0.4729	0.2515
	4	0.3422	0.4179	0.2810	0.5251	0.3624	0.0587
	5	0.2738	0.2856	0.2342	0.4014	0.2679	0.0587
OS	1	0.9773	0.9387	0.7784	0.9630	0.8938	0.7976
	2	0.9540	0.8415	0.6393	0.9466	0.7679	0.6155
	3	0.9282	0.7764	0.5342	0.9098	0.6702	0.5831
	4	0.8895	0.6790	0.4953	0.8979	0.5691	0.4574
	5	0.8895	0.6422	0.3698	0.8979	0.4791	0.3136
	**MMRF data**						
	**R-ISS (*n* = 658)**			**CRSS (*n* = 800)**		
	Year	1	2	3	1	2	3
PFS	1	0.9033	0.8132	0.6358	0.9325	0.8367	0.6611
	2	0.7957	0.6261	0.4040	0.8162	0.6734	0.4423
	3	0.6295	0.4862	0.3059	0.7008	0.5084	0.3129
	4	0.4641	0.3414	0.2781	0.5151	0.3711	0.2249
	5	0.2769	0.2450	0.2781	0.4121	0.2637	0.1799
OS	1	0.9807	0.9092	0.8559	0.9869	0.9379	0.8231
	2	0.9612	0.8372	0.6460	0.9689	0.8772	0.6780
	3	0.9286	0.7799	0.5211	0.9478	0.8217	0.5814
	4	0.8833	0.7461	0.4904	0.9478	0.7844	0.5293
	5	0.5748	0.7108	0.3678	0.9478	0.6569	0.4691

The 5-year OS for the MMIn (*n* = 384) was 89.79% for CRSS-1, 47.91% for CRSS-2, and 31.36% for CRSS-3 (**Table 3**). Overall, there is a substantial difference in the percentages of the 5-year OS and median OS for different risk groups which indicate that the groups were significant. A similar stratification was achieved when the CRSS model was applied on the MMRF test dataset. The 5-year OS for MMRF data was 94.78% for CRSS-1, 65.69% for CRSS-2, and 46.91% for CRSS-3 which is quite comparable to that obtained in the MMIn data. Higher values of C-index and hazard ratios as well as lower values of partial AIC and BIC on both datasets were indicative of the superior performance of our AI-based CRSS method as compared with R-ISS.

### Statistical Analysis on the Parameters Used in CRSS

The Kruskal–Wallis test was performed to compare the median values of the parameters age, albumin, β2M, calcium, eGFR, and hemoglobin across the three risk groups for both the MMIn and MMRF datasets. There was a significant increase (*p* < 0.05) in the values of age and β2M, while there was a significant decrease (*p* < 0.05) in the values of albumin, eGFR, and hemoglobin as the risk of disease increased (**Figures S5, S6**) for both the MMIn and MMRF datasets. Wilcoxon rank-sum test was performed to compare the median values of the parameters between two successive risk groups and showed significant variation of parameters for both datasets.

### Model Interpretation

**Figure 3:**
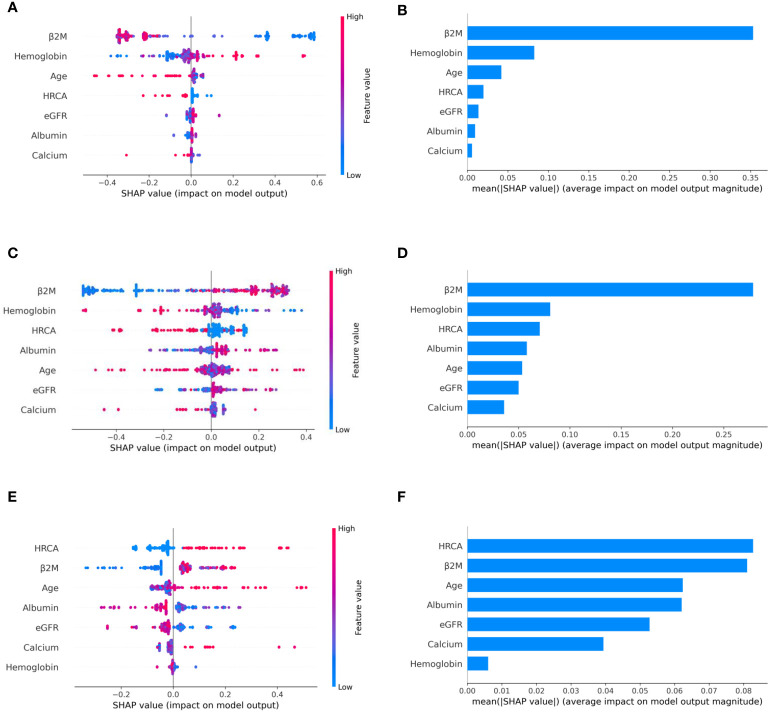
Model interpretation using SHAP (SHapley Additive exPlanations). SHAP summary plots for different risk stages inferred from MMIn data showing the relative impact of different parameters (top to bottom) contributing to a particular risk stage prediction. **(A, B)** CRSS-1: Normal levels of β2M and hemoglobin are the key contributors to the low-risk stage prediction. Furthermore, high values of age on the left side of the summary plot are pushing the model away from the low-risk prediction and are indicative of either intermediate or high risk. Overall, β2M has the highest impact and calcium has the lowest impact on the low-risk stage prediction. **(C, D)** CRSS-2: β2M and hemoglobin are the key contributors to the intermediate-risk stage. Elevated levels of β2M with lower levels of hemoglobin are indicative of intermediate risk. **(E, F)** CRSS-3: Presence of HRCA is contributing the most to the high-risk stage. Elevated values of β2M and calcium and lower levels of albumin, hemoglobin, and eGFR are contributing toward the high-risk stage prediction.

**Figure 4:**
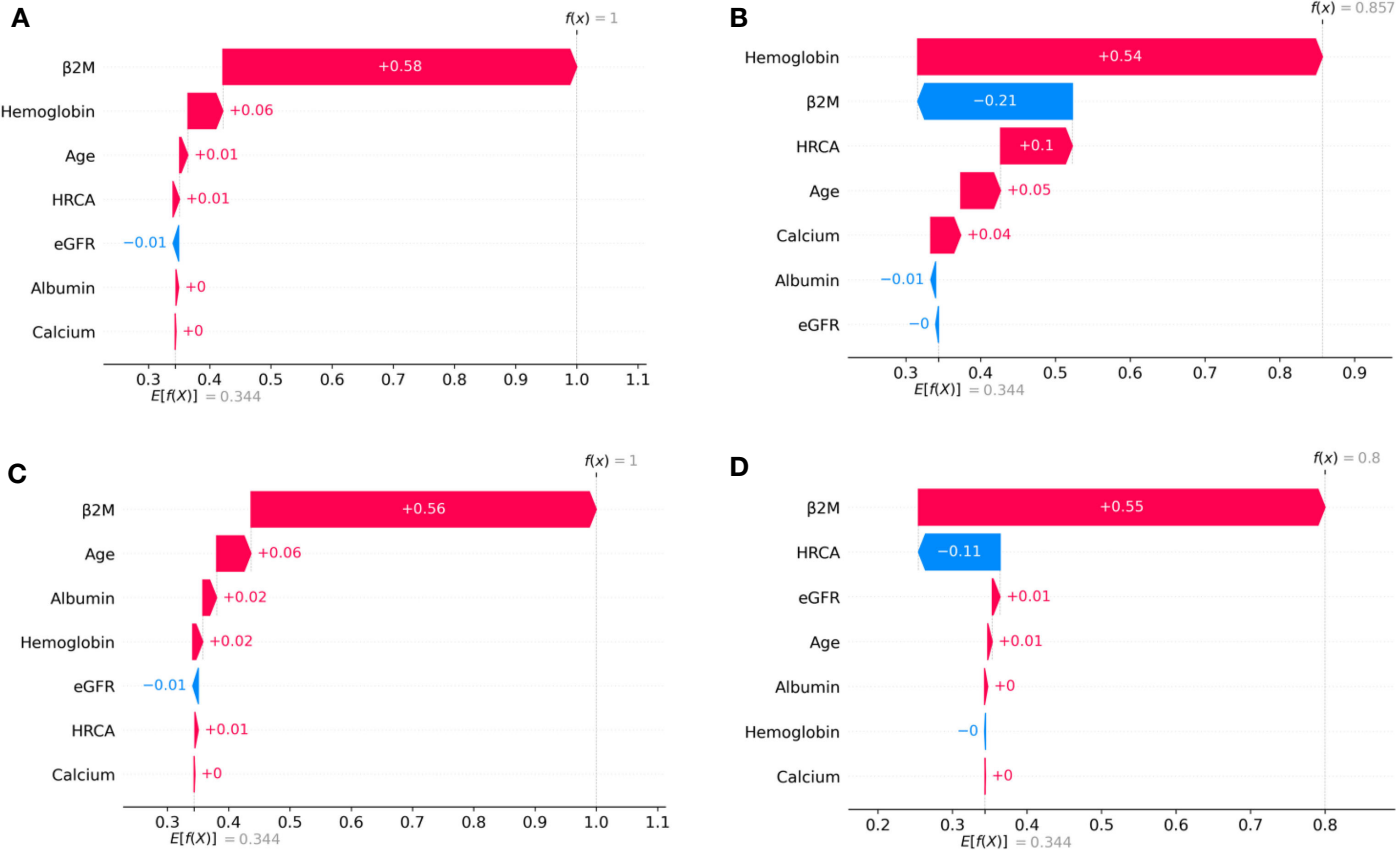
SHAP waterfall plots for the randomly chosen four patients in low-risk stage (CRSS-1) from the MMIn dataset. The pink color shows the positive impact of the feature, while the blue color shows the negative impact of the feature. Features with a positive impact contributed to the class of low-risk stage prediction, while features with a negative impact contributed to class opposite to low risk. β2M, hemoglobin, age, and HRCA have the highest overall impact on low-risk stage prediction in the MMIn dataset. However, this ranking itself differs from patient to patient as can be seen in **(A–D)**. **(A)** β2M has the highest impact followed by hemoglobin, age, and HRCA. **(B)** Hemoglobin has the highest impact followed by β2M and age. **(C, D)** β2M has the highest impact followed by age and HRCA.

**Figure 5:**
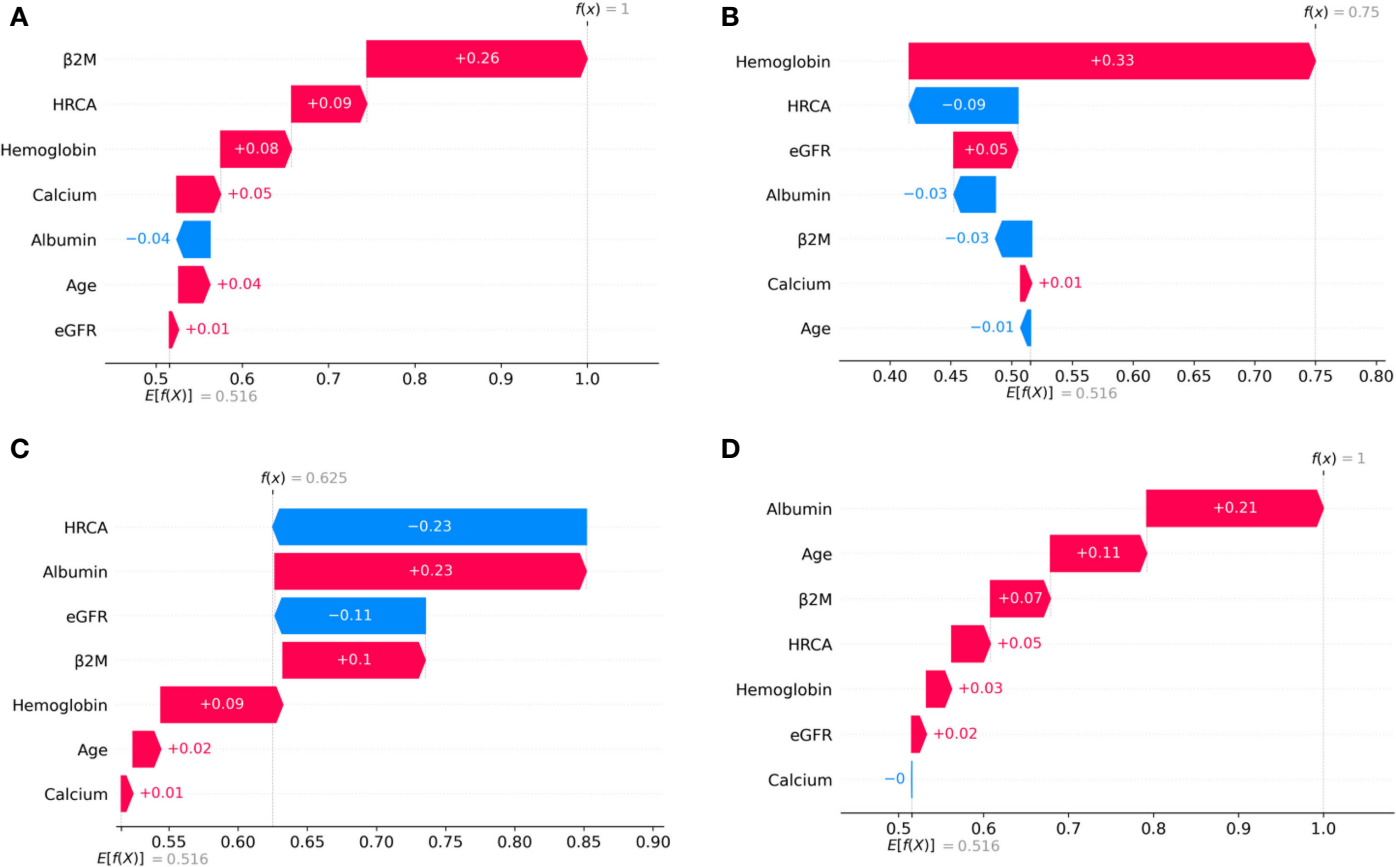
SHAP waterfall plots for the randomly chosen four patients in the intermediate-risk stage (CRSS-2) from the MMIn dataset. The pink color shows the positive impact of the feature, while the blue color shows the negative impact of the feature. Features with a positive impact contributed to the class of intermediate-risk stage prediction, while features with a negative impact contributed to the class opposite to intermediate risk. β2M, hemoglobin, HRCA, and albumin have the highest overall impact on the intermediate-risk stage prediction in the MMIn dataset. However, the ranking of the features itself differs from patient to patient as can be seen in **(A–D)**. **(A)** β2M has the highest impact followed by HRCA. **(B)** Hemoglobin has the highest impact followed by HRCA. **(C)** HRCA has the highest impact followed by albumin. **(D)** Albumin has the highest impact followed by age.

**Figure 6:**
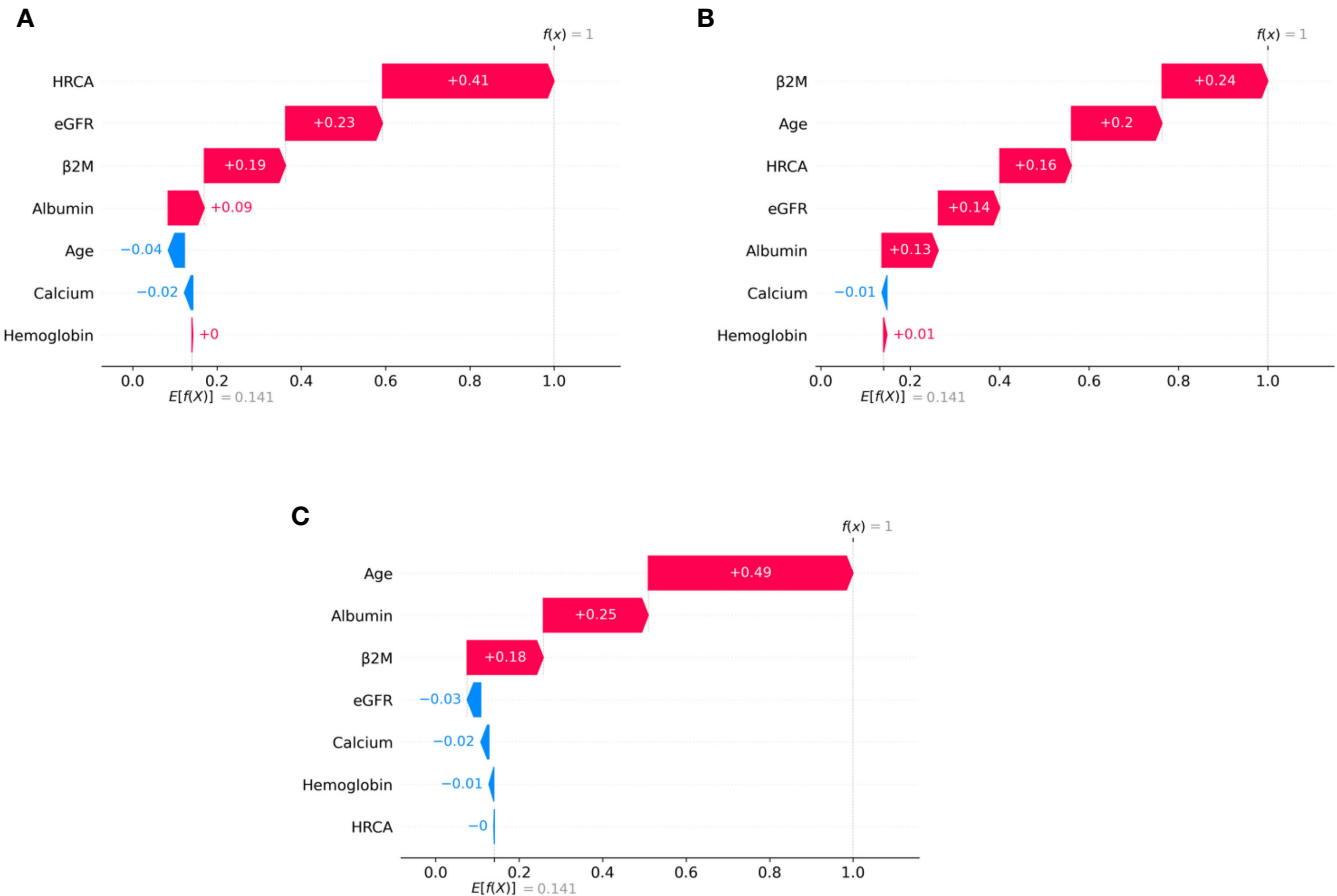
SHAP waterfall plots for randomly chosen patients in high-risk stage (CRSS-3) from the MMIn dataset. The pink color shows the positive impact of the feature, while the blue color shows the negative impact of the feature. Features with a positive impact contributed to the class of high-risk stage prediction, while features with a negative impact contributed to class opposite to highest risk. HRCA, β2M, age, and albumin have the highest overall impact on high-risk stage prediction. However, this ranking differs from patient to patient as can be seen in **(A–C)**. **(A)** HRCA has the highest impact. **(B)** β2M has the highest impact. **(C, D)** Age and albumin have the highest impact.

**Figure 7:**
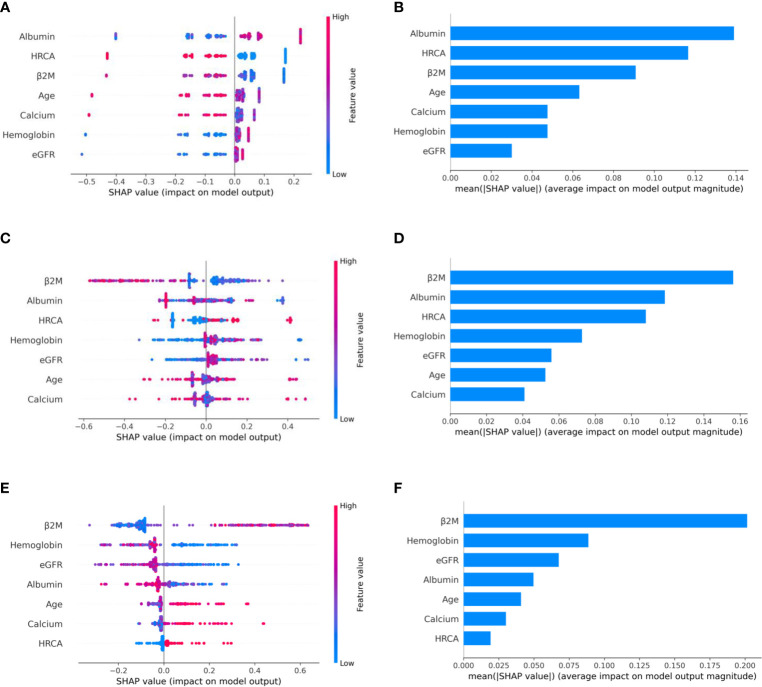
Model interpretation using SHAP. SHAP summary plots for different risk stages inferred in MMRF data showing the impact of different parameters used in the model. **(A, B)** CRSS-1: albumin, HRCA, and β2M have the highest impact on the low-risk stage. Normal levels of albumin, absence of HRCA, and lower values of β2M are contributing to low risk (CRSS-1) in myeloma patients. **(C, D)** CRSS-2: β2M, albumin, and HRCA are the key contributors to the intermediate-risk stage. **(E, F)** CRSS-3: β2M and hemoglobin have the highest impact on the high-risk stage. Elevated levels of β2M and lower values of hemoglobin are contributing toward the high-risk stage in the patient. Lower values of albumin and eGFR are further promoting high-risk stage prediction.

To ascertain the impact of individual parameters on risk stage predictions by CRSS, decision tree models built using the MMIn and MMRF datasets were analyzed using SHAP (**Figures 3**, **7**). Key contributors of high-risk predictions in the MMIn dataset were the presence of HRCA, elevated levels of β2M, higher age, and lower levels of albumin (**Figure 3**). Furthermore, lower levels of eGFR and hemoglobin along with elevated levels of calcium also contributed to high-risk prediction in the patients. It was observed from the waterfall plots (**Figures 4**–**6**) of the randomly chosen patients in different risk stages that the order of the impact of the parameters varied in different patients within the same risk category. For the high-risk category (**Figure 6**), HRCA had the highest impact on one of the randomly chosen patients; in another patient, β2M had the highest impact in contributing to high risk, while in the third patient, age and albumin had the highest prognostic impact. This suggests that the risk assessment in MM is a cumulative function of multiple factors. An individual parameter cannot adequately capture the risk associated with MM given that other prognostic parameters could influence the outcome. Furthermore, the complex association among different parameters that encapsulates the disease risk varies according to the patients, thereby leading to a varying order of impact of parameters in the patients. Hence, the AI-based decision tree algorithms can handle such an integrated analysis. This analysis reveals that each patient is unique and multiple factors interact and impact the outcome differently in individual patients.

## Discussion

The influence of ethnicities on clinical characteristics in patients belonging to distinct ethnic groups is well known, and therefore, it is of paramount interest to integrate the ethnic group-specific information in risk-staging models as it can affect the risk score prediction. The R-ISS (3) is the current standard of care for staging myeloma patients which includes a few HRCA, but molecular aberrations such as 1q gain and chromothripsis associated with adverse outcome have been overlooked (24). In fact, it includes t(4;14), which has lost significance in patients treated with triplet regimens (25). Besides, the R-ISS does not include any ethnic-specific information and, therefore, is not robust considering the large heterogeneous population of MM patients globally. An ideal risk-staging system would be based on all the known adverse prognostic factors including clinical, ethnic, and molecular aberrations. There is a tremendous heterogeneity in global healthcare systems that limit the availability of high-end molecular testing for all patients, and yet, the internet/electronic connectivity allows patients to receive medical advice from global leaders in medicine. Recently, an AI-supported risk-staging model, MRS (26), has been developed for NDMM; however, it does not include HRCA and ethnicity information. Considering the present world scenario, it is, thus, desirable to develop a simple risk-staging model that integrates ethnic-specific characteristics of the prognostic parameters that are easy to acquire in the healthcare settings worldwide.

### Risk-Staging Models and Their Performance as Compared With the R-ISS

In contrast to the R-ISS which utilizes four parameters, seven parameters were taken into consideration for designing the CRSS. It was observed that the cutoff values for these parameters derived using KAP vary in the two cohorts, one of which belongs to Indian and the other belongs to the American population. For the Indian data, there was a change in the cutoff values for β2M, age, eGFR, and hemoglobin, while there was no change in the cutoff value for calcium and albumin as shown in **Table 1**. For the MMRF data, there was a change in cutoff values for calcium, eGFR, hemoglobin, and age, while the cutoff values for albumin and β2M remain unchanged. The median age of onset of MM in the Indian population is almost a decade early as compared with the population in the USA (27, 28). This supported our assertion of choosing different cutoffs of age for MMIn from the MMRF dataset.

Various models were built on the different combinations of the parameters using both the established and proposed cutoffs for the two datasets. The best staging model for both datasets was obtained when the proposed cutoffs for the respective cohorts were used. When the ML-derived cutoffs were used for the parameters age, eGFR, hemoglobin, and β2M in the A3 model, performance was enhanced significantly in terms of high C-index and hazard ratios as compared with the R-ISS. A similar observation was noticed in the M4 model which utilized ML-derived cutoffs obtained for the MMRF dataset and achieved the best performance among all the models with a significant improvement in the C-index as well as hazard ratios as compared with the R-ISS. Overall, A3 and M4 were the best staging models for the MMIn and MMRF data, respectively. The improvement in the performance of the model verified our hypothesis that the cutoffs of the different parameters vary with different ethnicities.

The plausibility of the proposed model was further substantiated by performing significance testing. The Kruskal–Wallis test showed statistically significant variations (*p* < 0.05) in the median values of the parameters age, albumin, β2M, eGFR, and hemoglobin across the three risk groups (**Figures S4, S5**) for both datasets. Furthermore, the Wilcoxon rank-sum test revealed statistically significant variations (*p* < 0.05) in the median values of the parameters between two successive risk groups (CRSS-1 and CRSS-2; CRSS-2 and CRSS-3). Furthermore, CRSS for the MMIn and MMRF datasets were interpreted using SHAP (13) to establish the clinical relevance of the risk stages predicted by the CRSS. For the MMIn data, elevated levels of β2M and calcium with lower levels of eGFR and hemoglobin contributed to high risk, whereas in the MMRF data, elevated levels of β2M and lower levels of hemoglobin, eGFR, and albumin contributed to high risk in myeloma patients. These findings are in accordance with the observations mostly identified in high-risk MM patients. Additionally, it was observed that the order of impact of hemoglobin was higher in low-risk stage prediction in the MMIn dataset as compared with the MMRF dataset, while the order of impact of hemoglobin was higher in high-risk stage prediction in the MMRF dataset as compared with the MMIn dataset (**Figures 3**, **7**). The difference in the rankings can be attributed to the varying ethnicities and further confirmed our claim of using ethnicity-aware risk-staging models for MM. In the present study, we have used the MMIn and MMRF cohorts belonging to Indian and American ethnicities, respectively, for building CRSS models. Results on both cohorts have strengthened our claim that the robustness of the staging model is amplified by inclusion of ethnicity-specific cutoffs of the prognostic factors as well as by utilizing AI techniques.

The classification rules were obtained using a decision tree classifier on the classification output of the best performing models in both MMIn and MMRF data. Overall classification accuracy was 94.79% and 98% for the MMIn and MMRF data, respectively. Final risk stages were evaluated using the classification rules in both datasets. Furthermore, it is evident from the UMAP plots that both the MMIn and MMRF data were not visible as three separate risk groups initially in the absence of CRSS risk labels (**Figures S3A, C, E**). With the addition of these risk labels with every patient sample, the subjects could be seen to be grouped separately (where a group corresponds to one risk label) in the UMAP plot (**Figures S3B, D**). This demonstrates the ability of the CRSS model in identifying the risk groups correctly from the non-separable data. To further validate our model, we found risk stages in 123 prospective subjects of MMIn data that were not used to build the CRSS model. UMAP plots (**Figure S3F**) suggest that the prospective subjects got correctly aligned to their respective risk stages inferred *via* CRSS.

For the MMIn data, β2M was in the highest level of hierarchy in the classification rules followed by hemoglobin and HRCA (**Figure S2A**). For the MMRF data, the prognostic factor in the highest level of hierarchy was β2M followed by albumin and Hb (**Figure S2B**). The cutoff values for β2M, albumin, and Hb were 5.2, 3.55, and 9.64. The cutoffs for β2M and albumin were not changed, but the cutoff value proposed for Hb was 9.59, which was close to the observed value in the classification rules. This observation further justified our choice of using new cutoffs for the risk-staging model.

### Conclusion

In this work, we examined the impact of ethnicity-based cutoffs of laboratory parameters derived using the ML algorithm on risk prediction in Indian and American patients with MM. We trained different risk-staging models for both the MMRF and MMIn datasets. The best predictor model was obtained when ethnicity-specific cutoffs of the clinical parameters were utilized. Furthermore, we presented a new reliable and robust AI-enabled risk-staging system, namely, CRSS, which utilizes easily acquirable laboratory and clinical parameters, i.e., age, albumin, β2M, calcium, eGFR, and hemoglobin along with HRCA (**Table S5**). Risk stratification achieved by AI-assisted CRSS is able to better separate the patients into different risk groups as compared with the R-ISS. High concordance-index and hazard ratios reveal the superior performance of the CRSS as compared with the R-ISS.

Furthermore, the clinical and biological significance of the decision tree classifier rules for risk stage prediction in MM patients was deduced *via* SHAP analysis on both datasets. The successful evaluation of our proposed staging system on both datasets establishes the utility of the proposed ethnicity-aware staging system for NDMM patients, treated largely with novel agents or a combination thereof, in a real-world scenario. Our study also highlights the importance of application of AI in building CRSS, thereby enhancing the prediction of survival outcome and separability of risk stages in NDMM patients. We have also developed a web platform-based AI-assisted ethnicity-aware MM risk-staging calculator.

### Limitations and Future Work

The CRSS has been built on a smaller set of NDMM patients as compared with the R-ISS (3) study. In the future, the CRSS model may be tested on larger datasets with varying ethnic groups as the cohort size of the present study is 25% of the cohort used in the R-ISS reported in 2015. As the CRSS calculator becomes available online, data could be generated by independent groups for further validation in real-world scenarios.

## Data Availability Statement

The original contributions presented in the study are included in the article/**Supplementary Material**. Further inquiries can be directed to the corresponding authors. CRSS calculator can be found at: http://sbilab.iiitd.edu.in/pub_files/CRRScalculator_edit.html.

## Ethics Statement

The studies involving human participants were reviewed and approved by IEC, AIIMS. The patients/participants provided their written informed consent to participate in this study.

## Author Contributions

AF: methodology, software, formal analysis, investigation, validation, and writing—original draft preparation. AG: methodology, investigation, validation, writing—original draft preparation, resources, project management, and supervision. KS: formal analysis, validation, and supervision. LK: resources. AS: resources. RG: conceptualization, investigation, validation, resources, writing—original draft preparation, project management, and supervision. All the authors had full access to the final version of the report. All authors contributed to the article and approved the submitted version.

## Funding

This work was supported by grants from the Department of Biotechnology, Govt. of India (Grant: BT/MED/30/SP11006/2015), and the Department of Science and Technology, Govt. of India (Grant: DST/ICPS/CPS-Individual/2018/279(G)). The funding bodies had no role in study design, data collection, data analysis, data interpretation, or writing of the report.

## Conflict of Interest

The authors declare that the research was conducted in the absence of any commercial or financial relationships that could be construed as a potential conflict of interest.

## Publisher’s Note

All claims expressed in this article are solely those of the authors and do not necessarily represent those of their affiliated organizations, or those of the publisher, the editors and the reviewers. Any product that may be evaluated in this article, or claim that may be made by its manufacturer, is not guaranteed or endorsed by the publisher.

## References

[1] Durie BG Salmon SE. A Clinical Staging System for Multiple Myeloma Correlation of Measured Myeloma Cell Mass With Presenting Clinical Features, Response to Treatment, and Survival. Cancer (1975) 36(3):842–54. doi: 10.1002/1097-0142(197509)36:3<842::AID-CNCR2820360303>3.0.CO;2-U 1182674

[2] Greipp PR SanMiguel J Durie BG Crowley JJ Barlogie B Boccadoro M. International Staging System for Multiple Myeloma. J Clin Oncol (2005) 23:3412–20. doi: 10.1200/JCO.2005.04.242 15809451

[3] Palumbo A Avet-Loiseau H Oliva S Lokhorst HM Goldschmidt H Rosinol L. Revised International Staging System for Multiple Myeloma: A Report From International Myeloma Working Group. J Clin Oncol (2015) 33(26):2863. doi: 10.1200/JCO.2015.61.2267 26240224 PMC4846284

[4] Rago A Grammatico S Za T Levi A Mecarocci S Siniscalchi A. Prognostic Factors Associated With Progression of Smoldering Multiple Myeloma to Symptomatic Form. Cancer (2012) 118(22):5544–9. doi: 10.1002/cncr.27657 22786730

[5] Schinke M Ihorst G Duyster J Wäsch R Schumacher M Engelhardt M. Risk of Disease Recurrence and Survival in Patients With Multiple Myeloma: A German Study Group Analysis Using a Conditional Survival Approach With Long-Term Follow-Up of 815 Patients. Cancer (2020) 126(15):3504–15. doi: 10.1002/cncr.32978 32459378

[6] Howlader N Noone AM Krapcho M Miller D Bishop K Kosary CL. SEER Cancer Statistics Review, 1975–2014. (2017), based on November 2016 SEER data submission.

[7] Ailawadhi S Aldoss IT Yang D Razavi P Cozen W Sher T. Outcome Disparities in Multiple Myeloma: A SEER-Based Comparative Analysis of Ethnic Subgroups. Br J Haematol (2012) 158(1):91–8. doi: 10.1111/j.1365-2141.2012.09124.x 22533740

[8] Waxman AJ Mink PJ Devesa SS Anderson WF Weiss BM Kristinsson SY. Racial Disparities in Incidence and Outcome in Multiple Myeloma: A Population-Based Study. Blood (2010) 116(25):5501–6. doi: 10.1182/blood-2010-07-298760 PMC3031400 20823456

[9] Costa LJ Brill IK Omel J Godby K Kumar SK Brown EE. Recent Trends in Multiple Myeloma Incidence and Survival by Age, Race, and Ethnicity in the United States. Blood Adv (2017) 1(4):282–7. doi: 10.1182/bloodadvances.2016002493 PMC5727774 29296944

[10] Derman BA Jasielec J Langerman SS Zhang W Jakubowiak AJ Chiu BC. Racial Differences in Treatment and Outcomes in Multiple Myeloma: A Multiple Myeloma Research Foundation Analysis. Blood Cancer J (2020) 10(8):1–7. doi: 10.1038/s41408-020-00347-6 32770051 PMC7414120

[11] Yellapragada SV Fillmore NR Frolov A Zhou Y Dev P Yameen H. Vitamin D Deficiency Predicts for Poor Overall Survival in White But Not African American Patients With Multiple Myeloma. Blood Adv (2020) 4(8):1643. doi: 10.1182/bloodadvances.2019001411 32315398 PMC7189302

[12] Alexander DD Mink PJ Adami HO Cole P Mandel JS Oken MM. Multiple Myeloma: A Review of the Epidemiologic Literature. Int J Cancer (2007) 120(S12):40–61. doi: 10.1002/ijc.22718 17405120

[13] Lundberg SM Lee S-I. A Unified Approach to Interpreting Model Predictions. In: Proceedings of the 31st International Conference on Neural Information Processing Systems (Nips’17). Red Hook, NY, USA: Curran Associates Inc. (2017). p. 4768–77.

[14] Farswan A Gupta A. TV-DCT: Method to Impute Gene Expression Data Using DCT Based Sparsity and Total Variation Denoising, in: InICASSP 2019-2019 IEEE International Conference on Acoustics, Speech and Signal Processing (ICASSP), 2019 May 12. pp. 1244–8, IEEE.

[15] Gehlot S Farswan A Gupta A Gupta R. CT-NNBI: Method to Impute Gene Expression Data Using DCT Based Sparsity and Nuclear Norm Constraint With Split Bregman Iteration, in: In2019 IEEE 16th International Symposium on Biomedical Imaging (ISBI 2019), 2019 Apr 8. pp. 1315–8, IEEE.

[16] Farswan A Gupta A Gupta R Kaur G. Imputation of Gene Expression Data in Blood Cancer and Its Significance in Inferring Biological Pathways. Front Oncol (2020) 9:1442. doi: 10.3389/fonc.2019.01442 31970084 PMC6960109

[17] Montealegre JR Zhou R Amirian ES Scheurer ME. Uncovering Nativity Disparities in Cancer Patterns: Multiple Imputation Strategy to Handle Missing Nativity Data in the Surveillance, Epidemiology, and End Results Data File. Cancer (2014) 120(8):1203–11. doi: 10.1002/cncr.28533 PMC3981927 24436157

[18] Gupta R Kaur G Kumar L Rani L Mathur N Sharma A. Nucleic Acid Based Risk Assessment and Staging for Clinical Practice in Multiple Myeloma. Ann Hematol (2018) 97(12):2447–54. doi: 10.1007/s00277-018-3457-8 30056581

[19] Kumar S Paiva B Anderson KC Durie B Landgren O Moreau P. International Myeloma Working Group Consensus Criteria for Response and Minimal Residual Disease Assessment in Multiple Myeloma. Lancet Oncol (2016) 17:e328–46. doi: 10.1016/S1470-2045(16)30206-6 27511158

[20] Florkowski CM Chew-Harris JS. Methods of Estimating GFR–different Equations Including CKD-EPI. Clin Biochem Rev (2011) 32(2):75. 21611080 PMC3100284

[21] Rajkumar SV. Multiple Myeloma: 2016 Update on Diagnosis, Risk-Stratification, and Management. Am J Hematol (2016) 91(7):719–34. doi: 10.1002/ajh.24402 PMC5291298 27291302

[22] Eo SH Kang HJ Hong SM Cho H. K-Adaptive Partitioning for Survival Data, With an Application to Cancer Staging. (2013). arXiv preprint arXiv:1306.4615.

[23] Monti S Tamayo P Mesirov J Golub T. Consensus Clustering: A Resampling-Based Method for Class Discovery and Visualization of Gene Expression Microarray Data. Mach Learn (2003) 52(1-2):91–118. doi: 10.1023/A:1023949509487

[24] Kaur G Gupta R Mathur N Rani L Kumar L Sharma A. Clinical Impact of Chromothriptic Complex Chromosomal Rearrangements in Newly Diagnosed Multiple Myeloma. Leukemia Res (2019) 76:58–64. doi: 10.1016/j.leukres.2018.12.005 30576858

[25] Avet-Loiseau H Leleu X Roussel M Moreau P Guerin-Charbonnel C Caillot D. Bortezomib Plus Dexamethasone Induction Improves Outcome of Patients With T (4; 14) Myeloma But Not Outcome of Patients With Del (17p). J Clin Oncol (2010) 28(30):4630–4. doi: 10.1200/JCO.2010.28.3945 20644101

[26] Farswan A Gupta A Gupta R Hazra S Khan S Kumar L. AI-Supported Modified Risk Staging for Multiple Myeloma Cancer Useful in Real-World Scenario. Trans Oncol (2021) 14(9):101157. doi: 10.1016/j.tranon.2021.101157 PMC8278429 34247136

[27] Unnikrishnan A Khan AM Narayan P Norkin M. Striking Age Differences of Multiple Myeloma (MM) Diagnosis in Patients of Indian and Pakistani Descent in the United States Compared to Native Countries. J Clin Oncol (2017) 35:e13070. doi: 10.1200/JCO.2017.35.15_suppl.e13070

[28] Konatam AM Sadashivudu G. Age of Onset of Multiple Myeloma: A Paradigm Shift in Indian Patients. Indian J Appl Res (2016) 6:3.

