# Supplementary material for: Does Ethnicity Matter in Multiple Myeloma Risk Prediction in the Era of Genomics and Novel Agents? Evidence From Real-World Data
Source: Front Oncol. 2021 Nov 9;11:720932. doi: 10.3389/fonc.2021.720932 (PMC8630746; doi:10.3389/fonc.2021.720932)
Supplement: Supplementary file 1 [file DataSheet_1.pdf]

Supplementary Material

1. Supplementary Data

1.1. Methods used for statistical analysis in the staging model

1.1.1. Kaplan-Meier (KM) method

KM method[1] is a non parametric method that is used for estimation of survival probability from the observed survival times like overall survival or progression free survival. The survival probability $S(t_k)$ at time t_k can be calculated as-

$$S(t_k) = S(t_{k-1})\left(1 - \frac{d_k}{n_k}\right)$$

Where, $S(t_{k-1})$ is the probability that an individual survives from the time point of the diagnosis of a disease to a time t_{k-1} , n_k is the number of patients at risk just before the time instant t_k, d_k is the number of events (death or disease progression) that have taken place till time t_k. Kaplan-Meier analysis was done to evaluate the survival curves of the high and low risk groups obtained from individual prognostic factors- albumin, β2M, calcium, eGFR, Hemoglobin, age and high risk cytogenetic abnormalities (HRCA). KM analysis was performed using the command- KaplanMeierFitter() available in python package "lifelines".[2]

1.1.2. Log Rank Test

Log rank test[3,4] is performed to check if the difference in the survival between high and low risk groups is significant or not. The null hypothesis of the test is that the survival curves for the two groups are identical. If the p-value of the test is below 0.05, null hypothesis is rejected and it is believed that there is significant difference between the survival patterns of the two groups. Log rank test was performed using the command- logrank_test() available in python package "lifelines".[2]

1.1.3. Cox Proportional hazard Method

Cox proportional hazard model[5] is used to examine the impact of predictor variables on survival. Cox model is expressed by the hazard function, $h(t)$, interpreted as the risk of an event(death or progression) in an individual at time t. $h(t)$ can be estimated as-

$$h(t) = h_0(t) \times exp(b_1 z_1 + b_2 z_2 + \ldots + b_n z_n)$$

Where t denotes the survival time, $h(t)$ is the hazard function computed on a set of n covariates z_1, z_2, ….z_n, coefficients b_1, b_2,....b_n quantify the impact of the covariate, n denotes the total number of covariates and $h_0(t)$is the baseline hazard. The quantities $exp(b_k)$ are called the

hazard ratios. A hazard ratio above 1 means that the covariate is conclusively associated with the probability of the event to occur and therefore has an opposite outcome on the duration of the survival.

We performed univariate as well as multivariate Cox hazard analysis in our study. For univariate analysis, the hazard function was given by -

$$h(t) = h_0(t) \times exp(b_1 z_1)$$

Where, $z_1$represents the different covariates- age, albumin, β2M, eGFR, calcium, hemoglobin and HRCA taken one at a time in the univariate analysis. Hazard ratio corresponding to each factor was computed individually for both progression free survival (PFS) and overall survival (OS).

For the multivariate analysis, the hazard function was given by-

$$h(t) = h_0(t) \times exp(b_1 z_1 + b_2 z_2 + b_3 z_3 + b_4 z_4 + b_5 z_5 + b_6 z_6 + b_7 z_7)$$

Where, covariates z_1, z_2, z_3, z_4, z_5, z_6, $z_7$denote the seven prognostics factors used in the staging model- age, albumin, β2M, eGFR, calcium, hemoglobin and HRCA respectively. Multivariate Cox hazard model was fitted on these parameters to evaluate the combined impact of all the parameters on the duration of the survival.

Cox Hazard analysis was done using the command- CoxPHFitter() available in python package “lifelines”.[2] Breslow estimation method has been used as the baseline method in Cox proportional hazard model.

1.1.4. Wilcoxon rank-sum test and Kruskal-Wallis test

Wilcoxon rank-sum test and Kruskal-Wallis test are non parametric tests used to compare samples of two groups and more than two groups respectively. Both these tests were used to determine if the difference in the median values of the individual parameters in the three risk stages is statistically significant or not.

1.1.5. SHAP (SHapley Additive exPlanations)

SHAP is essentially a technique based on game theory that facilitates the understanding of the output of any machine learning model. It helps in the interpretation of any machine learning model in a better way. ‘SHAP’ package is available in python.

1.2 Machine learning algorithms used in the staging model

1.2.1 K-adaptive partitioning

K-adaptive partitioning[6] utilizes a multi way partitioning algorithm to divide the data into K-subgroups based on the information obtained from a prognostic factor. In our work, we have used multiple prognostic factors to obtain an efficient partition for all of them. The algorithm is designed in a way such that the resulting subgroups show a significant difference in the survival patterns. KAP was performed in R using the package “kaps”.[6]

1.2.2 Gaussian Mixture Model (GMM) clustering

GMM[7] clustering is an unsupervised clustering algorithm to cluster data into distinct groups. A GMM model is a probabilistic model with the assumption that all the generated data points belong to a mixture of a finite number of Gaussian distributions with unknown parameters. It utilizes Expectation maximization algorithm to fit the mixture of gaussian models to the data. The function for GMM clustering is available in the module sklearn.cluster of python package “scikit-learn”.[8]

1.2.3 Agglomerative clustering

Agglomerative clustering is a type of hierarchical clustering that is used to group similar objects together in a cluster. It starts by treating each data point as an individual cluster and then it successively merges a pair of clusters based on a similarity score till the required number of clusters are generated. The function for agglomerative clustering is available in the module sklearn.cluster of python package “scikit-learn”.[8]

1.2.4 Decision Tree classifier

Decision tree classifier is a supervised method to generate a decision tree. It is an optimized version of the CART (Classification and Regression Trees) algorithm[9]. A decision tree is a flowchart like tree structure, where each node depicts a choice on an attribute/variable, each branch depicts the outcome of the choice and each leaf (terminal node) represents the label associated with the class. Classification rules were obtained in our study using the decision tree classifier in “scikit-learn” python package.[8]

References:

1. Kaplan EL, Meier P. Nonparametric estimation from incomplete observations. Journal of the American statistical association. 1958 Jun 1;53(282):457-81.
2. Davidson-Pilon C. lifelines: survival analysis in Python. Journal of Open Source Software. 2019 Aug 4;4(40):1317.
3. Mantel N. Evaluation of survival data and two new rank order statistics arising in its consideration. Cancer Chemother Rep. 1966 Mar 1;50(3):163-70.
4. Peto R, Peto J. Asymptotically efficient rank invariant test procedures. Journal of the Royal Statistical Society: Series A (General). 1972 Mar;135(2):185-98.
5. Cox DR. Regression models and life‐tables. Journal of the Royal Statistical Society: Series B (Methodological). 1972 Jan;34(2):187-202.
6. Eo SH, Kang HJ, Hong SM, Cho H. K-adaptive partitioning for survival data, with an application to cancer staging. arXiv preprint arXiv:1306.4615. 2013 Jun 19.
7. Rasmussen C. The infinite Gaussian mixture model. Advances in neural information processing systems. 1999;12:554-60.
8. Pedregosa F, Varoquaux G, Gramfort A, Michel V, Thirion B, Grisel O, Blondel M, Prettenhofer P, Weiss R, Dubourg V, Vanderplas J. Scikit-learn: Machine learning in Python. the Journal of machine Learning research. 2011 Nov 1;12:2825-30.
9. Breiman L, Friedman J, Stone CJ, Olshen RA. Classification and regression trees. CRC press; 1984.

2. Supplementary Tables and Figures

Table S1: Baseline demographic, laboratory and clinical characteristics of multiple myeloma (MM) patients of MMIn and MMRF cohort.

Parameters	MMIn (n=1070)	MMRF (n=900)
Age (Median, Range; in years)	56 (18-87)	62 (27 - 91)
Male/<br>Female	710 (66·36%)<br>360 (33·64%)	529 (58.78%)<br>371 (41.22%)
Hemoglobin (g/dL)<br><10<br>≥10	599 (55·98%)<br>471 (44·02%)	331 (36·77%)<br>569 (63·23%)
Serum albumin (g/dL)<br><3·5<br>≥3·5	449 (41·96%)<br>621 (58.04%)	328 (36·44%)<br>572 (63·56%)
Beta 2 microglobulin (mg/L)<br><5·5<br>≥5·5	534 (49.90%)<br>536 (50.09%)	661 (73·44%)<br>239 (26·56%)
Serum LDH (IU/L)<br>≤280<br>>280	929 (86·82%)<br>141 (13·18%)	850 (94·44%)<br>50 (5·56%)
Serum creatinine (mg/dL)<br>≤2<br>>2	830 (77.57%)<br>240 (22.43%)	816 (90·66%)<br>84 (9·34%)
Serum calcium (mg/dL)<br>≤11<br>>11	935 (87.38%)<br>135 (12.62%)	831 (92·33%)<br>69 (7·67%)
ISS 1/2/3	212/325/552	342/319/239
R-ISS 1/2/3	32/158/61	107/505/91

Table S2: The parameters of the two cohorts MMIn and MMRF were compared via unpaired Wilcoxon rank-sum test. If the p-value < 0.05, it can be concluded that the median is significantly different in both the cohorts. Median value of albumin was not statistically different between MMIn and MMRF, while these were statistically different for the rest of the parameters across the cohorts.

Parameter	*p*-value
age	3.09E-34
albumin	0.2
β2M	2.54E-34
calcium	0.00029
eGFR	1.98E-09
Hemoglobin	2.89E-34

Table S3: Univariate Cox hazard analysis on the prognostic factors- age, albumin, β2M, calcium, eGFR, hemoglobin and high risk cytogenetic abnormalities (HRCA). Hazard ratios of all the parameters except HRCA were calculated on the full data (*n*=1070 for MMIn and *n*=900 for MMRF). Hazard ratio of HRCA was found using data for which HRCA information was present (*n*=384 for MMIn and *n*=800 for MMRF).

**Parameter (lower risk threshold, higher risk threshold)**	**MMIn (*n*=1070, HRCA available for *n*=384)**							
	**PFS**				**OS**			
	**HR**	**CI lower limit**	**CI upper limit**	***p*-value**	**HR**	**CI lower limit**	**CI upper limit**	***p*-value**
**Age (67≤, >67)**	1.35	1.06	1.71	0.01	1.92	1.46	2.51	1.92e-06
**Albumin (>3·5, ≤ 3·5)**	1.13	0.96	1.32	0.11	1.41	1.15	1.73	8e-04
**β2M (<4·78, ≥ 4·78)**	1.64	1.4	1.93	1.34e-09	2.27	1.82	2.82	1.94e-13
**Calcium (<11, ≥ 11)**	1.36	1.08	1.69	0.008	1.50	1.13	1.98	3.9e-3
**eGFR (>48·2,≤48·2)**	1.19	1.00	1.41	0.04	1.50	1.21	1.85	1.7e-04
**Hb (>12·3, ≤ 12·3)**	1.6	1.26	2.03	1.00e-04	2.55	1.75	3.7	9.60e-07
**HRCA(del17, t(4;14), t(14;16))**	1.68	1.23	2.28	0.00085	1.9	1.29	2.8	0.00112
**Parameter (lower risk threshold, higher risk threshold)**	**MMRF (*n*=900, HRCA available for *n*=800)**							
	**PFS**				**OS**			
	**HR**	**CI lower limit**	**CI upper limit**	***p*-value**	**HR**	**CI lower limit**	**CI upper limit**	***p*-value**
**Age (69≤, >69)**	1.79	1.45	2.20	<5e-06	2.41	1.79	3.23	<5e-06
**Albumin (>3·5, ≤ 3·5)**	1.44	1.19	1.75	0.0002	2.06	1.53	2.76	<5e-06
**β2M (<5·5, ≥ 5.5)**	1.92	1.56	2.35	<5e-05	2.76	2.06	3.69	<5e-06
**Calcium (<10.52, ≥ 10.52)**	1.67	1.28	2.19	0.00017	2.24	1.58	3.18	1e-05
**eGFR (>48·3,≤48·3)**	1.91	1.53	2.38	<5e-05	2.57	1.90	3.49	<5e-06
**Hb (>9.59, ≤ 9.59)**	1.80	1.47	2.20	<5e-05	2.07	1.55	2.78	<5e-06
**HRCA(del17, t(4;14), t(14;16))**	1.08	0.87	1.35	0.48012	1.38	0.99	1.91	0.05388

Table S4: Multivariate Cox hazard analysis on the prognostic factors- age, albumin, β2M, calcium, eGFR, hemoglobin and high risk cytogenetic abnormalities (HRCA). Multivariate analysis was performed on data with HRCA information (*n*=384 for MMIn and *n*=800 for MMRF).

Parameter (lower risk threshold, higher risk threshold)	**MMIn (*n*=384)**							
	**PFS**				**OS**			
	**HR**	**CI Lower limit**	**CI Upper limit**	***p*-value**	**HR**	**CI Lower limit**	**CI Upper limit**	***p*-value**
**Age (67≤, >67)**	1.40	0.91	2.16	0.12657	2.63	1.67	4.15	0.00003
**Albumin (>3·5, ≤ 3·5)**	0.92	0.70	1.22	0.57215	0.96	0.67	1.39	0.83982
**β2M (<4·78, ≥ 4·78)**	1.57	1.14	2.15	0.00544	3.30	2.06	5.29	<5e-06
**Calcium (<11, ≥ 11)**	1.68	1.09	2.59	0.01841	1.34	0.72	2.48	0.35021
**eGFR (>48·2,≤48·2)**	0.91	0.66	1.25	0.56159	0.74	0.50	1.11	0.15055
**Hb (>12·3, ≤ 12·3)**	1.63	0.97	2.74	0.06395	1.84	0.82	4.11	0.14009
**HRCA(del17, t(4;14), t(14;16))**	1.48	1.08	2.03	0.01396	1.44	0.97	2.14	0.0739
**Parameter (lower risk threshold, higher risk threshold)**	**MMRF (*n*=800)**							
	**PFS**				**OS**			
	**HR**	**CI Lower limit**	**CI Upper limit**	***p*-value**	**HR**	**CI Lower limit**	**CI Upper limit**	***p*-value**
**Age (69≤, >69)**	1.52	1.20	1.92	0.00047	1.98	1.42	2.77	0.00006
**Albumin (>3·5, ≤ 3·5)**	1.23	0.98	1.54	0.06812	1.74	1.23	2.45	0.00179
**β2M (<5·5, ≥ 5.5)**	1.25	0.94	1.65	0.12029	1.48	1.00	2.20	0.04926
**Calcium (<10.52, ≥ 10.52)**	1.62	1.21	2.18	0.00136	1.94	1.29	2.90	0.00143
**eGFR (>48·3,≤48·3)**	1.19	0.89	1.60	0.24308	1.47	0.98	2.21	0.0645
**Hb (>9.59, ≤ 9.59)**	1.50	1.17	1.93	0.00134	1.35	0.93	1.96	0.1097
**HRCA(del17, t(4;14), t(14;16))**	1.11	0.89	1.39	0.34433	1.42	1.02	1.97	0.03786

Table S5: Parameters used in the staging systems- R-ISS and CRSS (proposed).

**Revised-ISS (R-ISS)**	**CRSS**
● Albumin<br>● Beta2microglobulin<br>● Cytogenetic abnormalities<br>● LDH	● Age<br>● Albumin<br>● Beta2microglobulin<br>● Calcium<br>● eGFR<br>● Hemoglobin<br>● High risk cytogenetic abnormalities

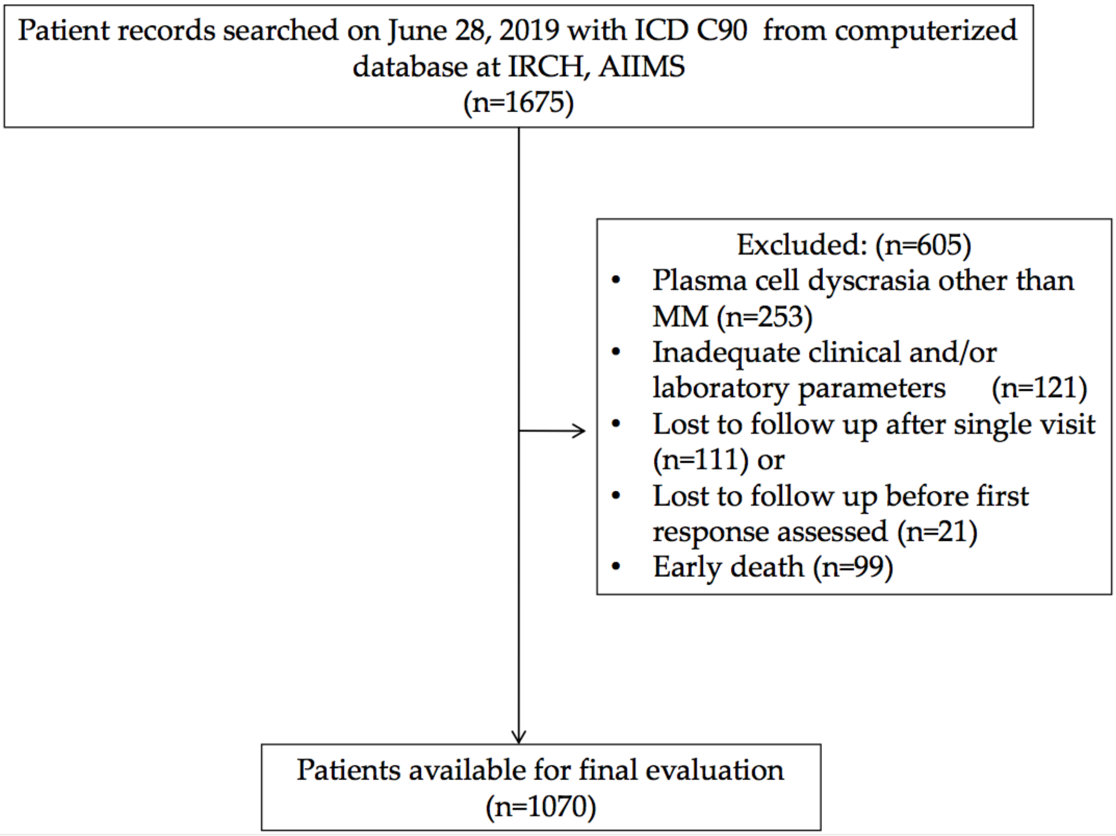

Figure S1: Flowchart of Study Population

A

B

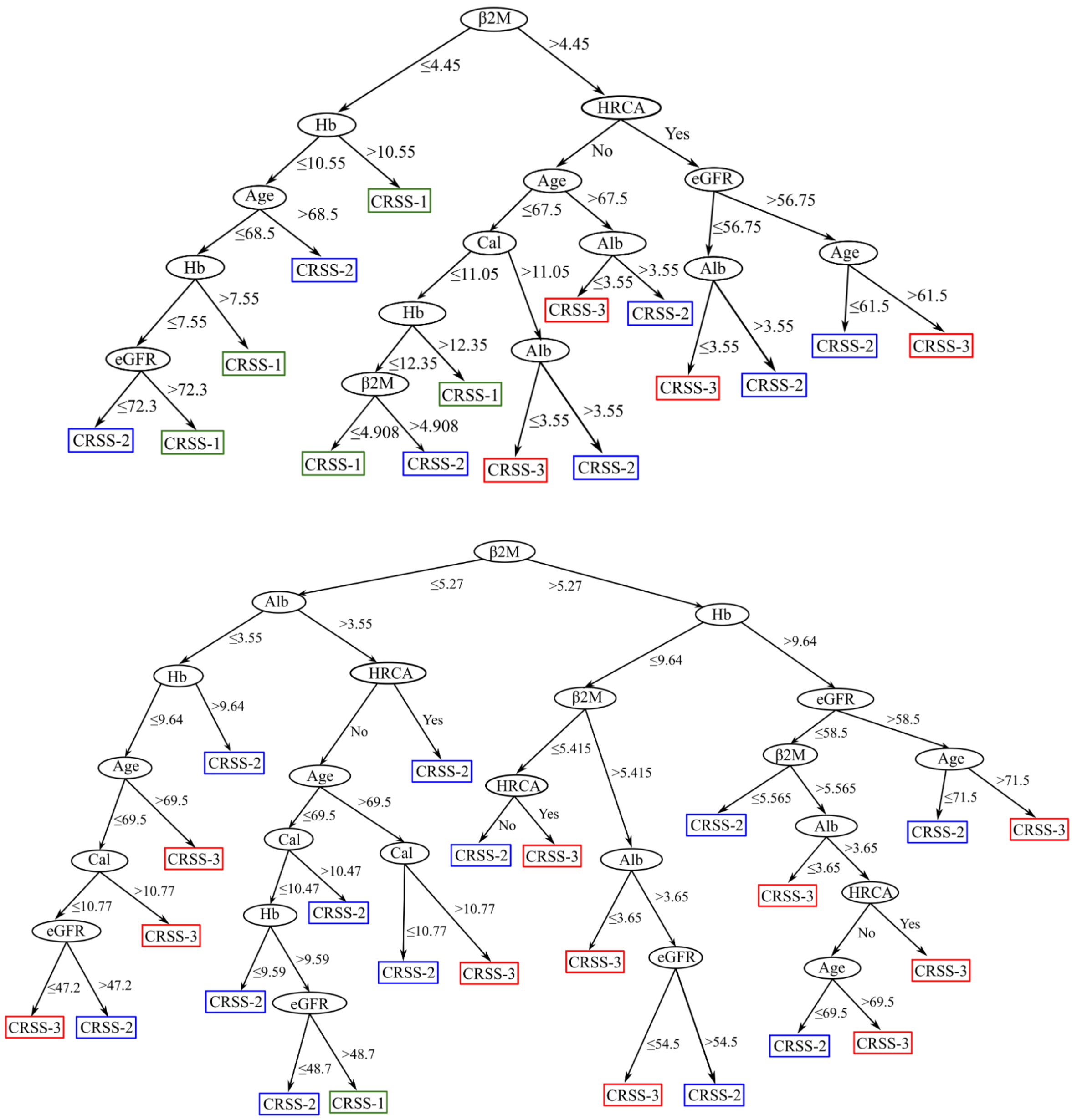

Figure S2: Hierarchical rule based tree structure to assign data samples to CRSS-1 (Low), CRSS-2 (Inter) and CRSS-3 (High) groups. Parameters: Age: Age; Alb: Albumin; β2M: beta2-microglobulin; Ca: Calcium; eGFR: estimated glomerular filtration rate; Hb: hemoglobin and CA: High risk cytogenetic abnormalities. A. MMIn cohort B. MMRF cohort.

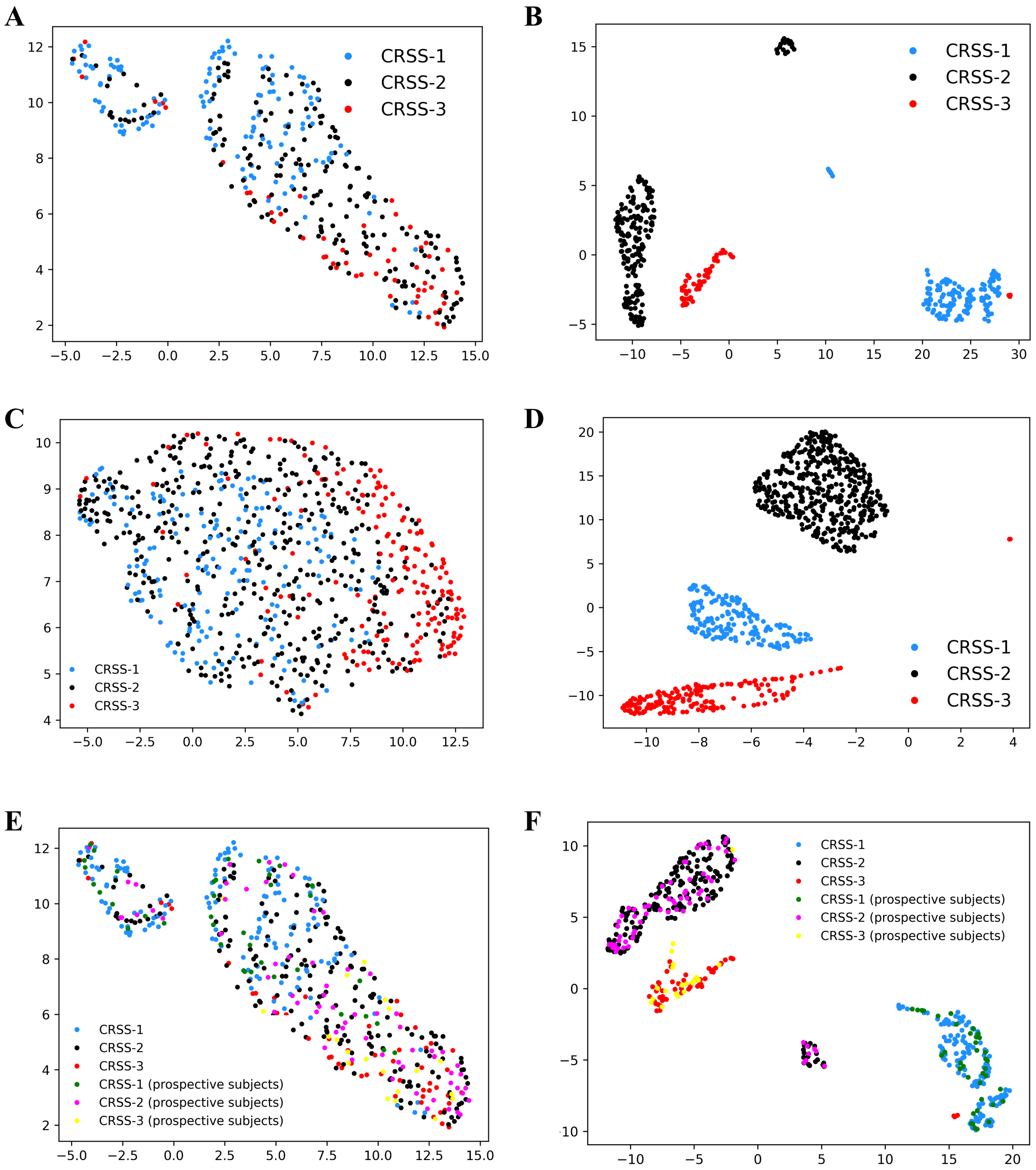

Figure S3: UMAP scatter plot of (A), (B) MMIn data and (C), (D) MMRF data depicting the data in absence and presence of risk stage labels respectively. The plot indicates that both the MMIn and MMRF data were not visible as three separate risk groups initially in the absence of CRSS risk labels. With the addition of these risk labels with every patient sample, the subjects are now grouped separately (where a group corresponds to one risk label) in the UMAP plot. This demonstrates the ability of the CRSS model in identifying the risk groups correctly from the non-separable data. Performance of the model was further validated by identifying risk stages in 123 prospective MMIn subjects that were not used to build CRSS. (E) UMAP scatter plot of the prospective MMIn subjects (n=123) along with the MMIn data of 384 patients reveals that data is not visible as separate risk groups in absence of risk stage labels and (F) UMAP scatter plot reveals that the prospective MMIn subjects align themselves to their respective risk groups after addition of risk stage labels. Prospective subjects are shown in green, magenta and yellow colors and perfectly maps to the CRSS-1 (shown by blue dots), CRSS-2 (shown by black dots) and CRSS-3 (shown by red dots) risk groups, respectively.

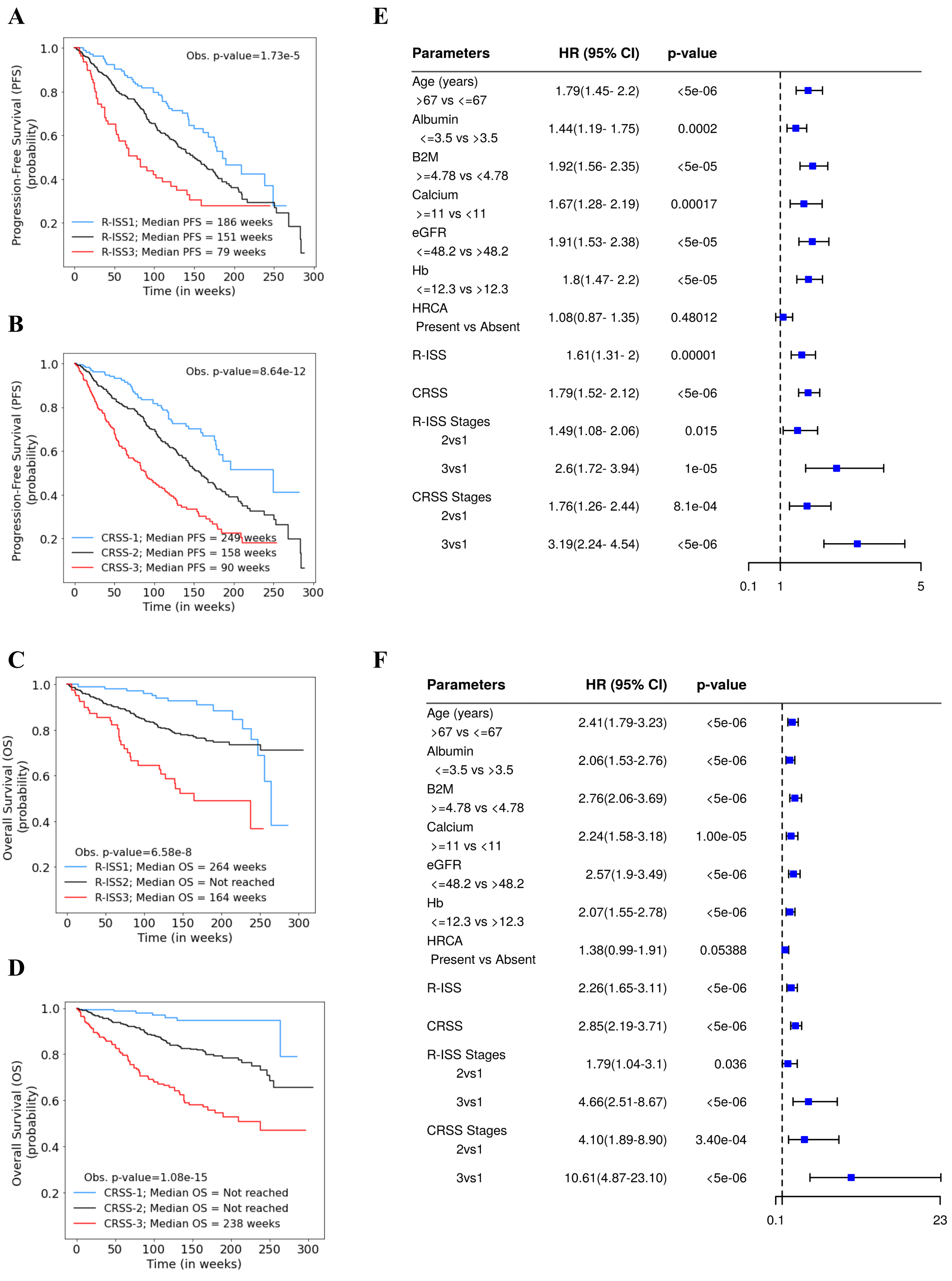

Figure S4: A, B - Progression-Free Survival in patients with MM from MMRF cohort (n=900) stratified by R-ISS (n=658) and the proposed CRSS (n=800) respectively. R-ISS1 is the low risk stage, R-ISS2 is the intermediate risk stage and R-ISS3 is the high risk stage. Median PFS for R-ISS1, R-ISS2 and R-ISS3 are 186, 151 and 79 weeks respectively. Observed p-value obtained after performing a log rank test on R-ISS is 1.73e-5.

Similarly, CRSS-1 is the low risk stage, CRSS-2 is the intermediate risk stage and CRSS-3 is the high risk stage. Median PFS for CRSS-1, CRSS-2 and CRSS-3 are 249, 158 and 90 weeks respectively. Observed p-value obtained after performing a log rank test on CRSS is 8.64e-12. C, D - Overall Survival in patients with MM from MMRF cohort (n=900) stratified by R-ISS (*n*=658) and the proposed CRSS (*n*=800) respectively. Median OS for R-ISS1, R-ISS2 and R-ISS3 are 264, Not reached and 164 weeks respectively. Observed p-value obtained after performing a log rank test on R-ISS is 6.58e-8. Median OS for CRSS-1, CRSS-2 and CRSS-3 are Not reached, Not reached and 238 weeks respectively. Observed p-value obtained after performing a log rank test on CRSS is 1.08e-15. E, F - Univariate Cox hazard analysis on the prognostic factors- age, albumin, β2M, calcium, eGFR, hemoglobin and high risk cytogenetic abnormalities (HRCA) for PFS and OS respectively. Hazard ratios for all the parameters except HRCA were calculated on complete data (*n*=900) for MMRF dataset. Hazard ratio for HRCA and the risk staging models were found using the data for which HRCA information was present (*n*=800 for MMRF dataset).

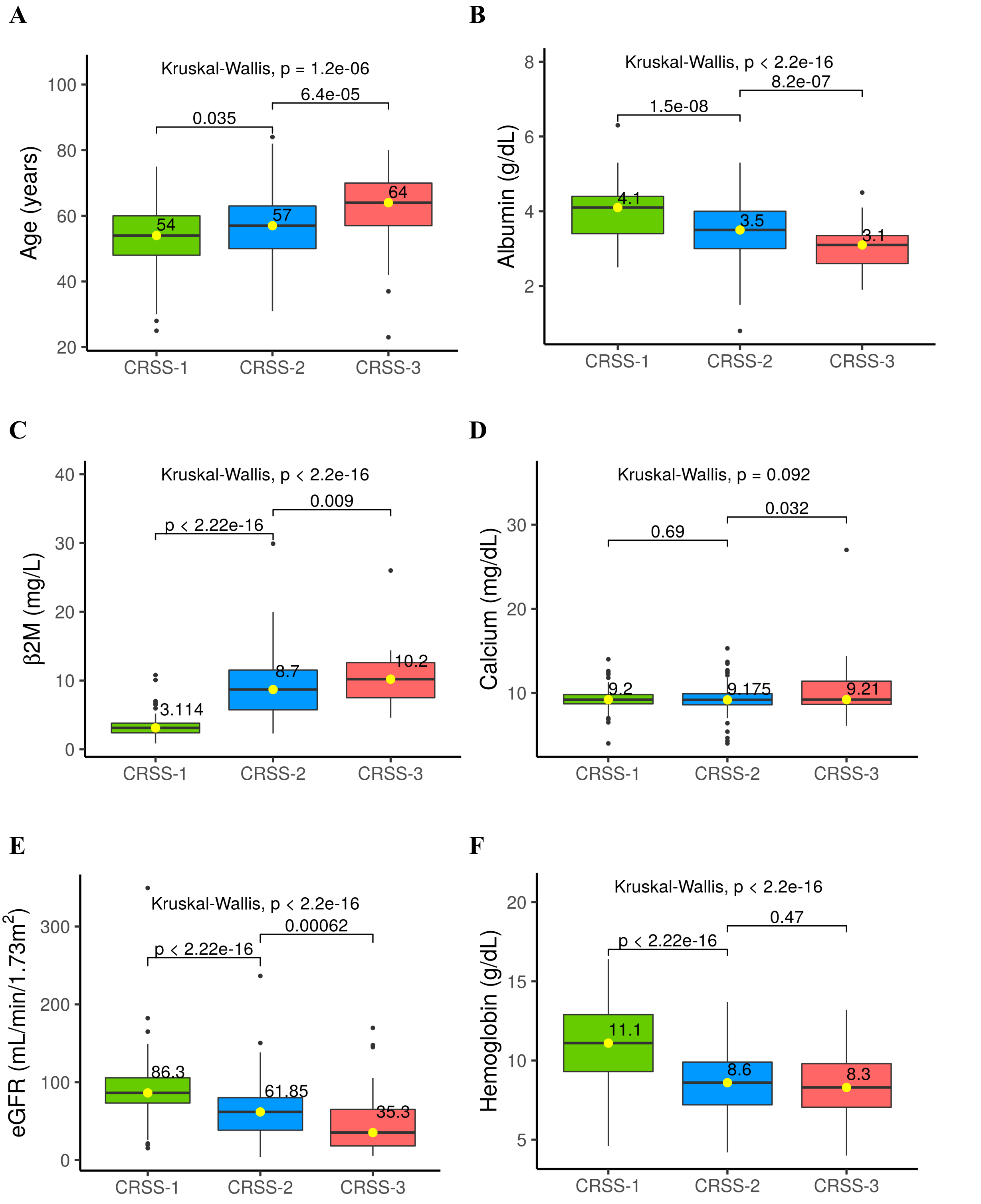

Figure S5: Boxplot showing the variation of the six parameters- A-age, B-albumin, C- β2M, D- calcium, E- eGFR and F-hemoglobin for MMIn dataset at CRSS-1, CRSS-2 and CRSS-3. The median values of all the parameters differ significantly across the three risk stages. Age and β2M are increasing while albumin, eGFR and hemoglobin are decreasing as the risk increases. Wilcoxon rank-sum test was used to compare two risk groups and Kruskal-Wallis test was used for comparing the three risk groups.

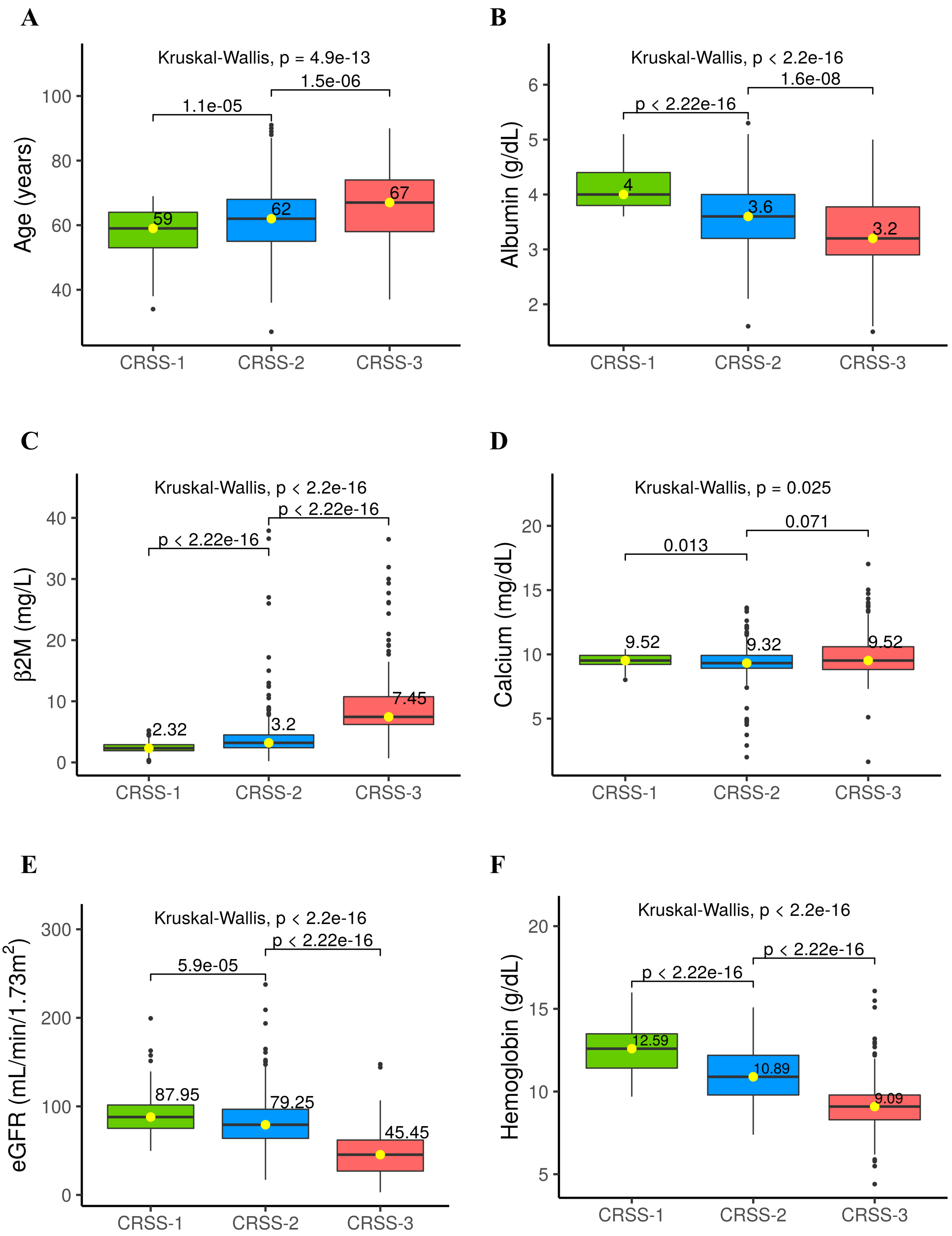

Figure S6: Boxplot showing the variation of the six parameters- A-age, B-albumin, C- β2M, D- calcium, E-eGFR and F-hemoglobin for MMRF dataset at CRSS-1, CRSS-2 and CRSS-3. The median values of all the parameters differ significantly across the three risk stages. Age and β2M are increasing while albumin, eGFR and hemoglobin are decreasing as the risk increases. Wilcoxon rank-sum test was used to compare two risk groups and Kruskal-Wallis test was used for comparing the three risk groups.

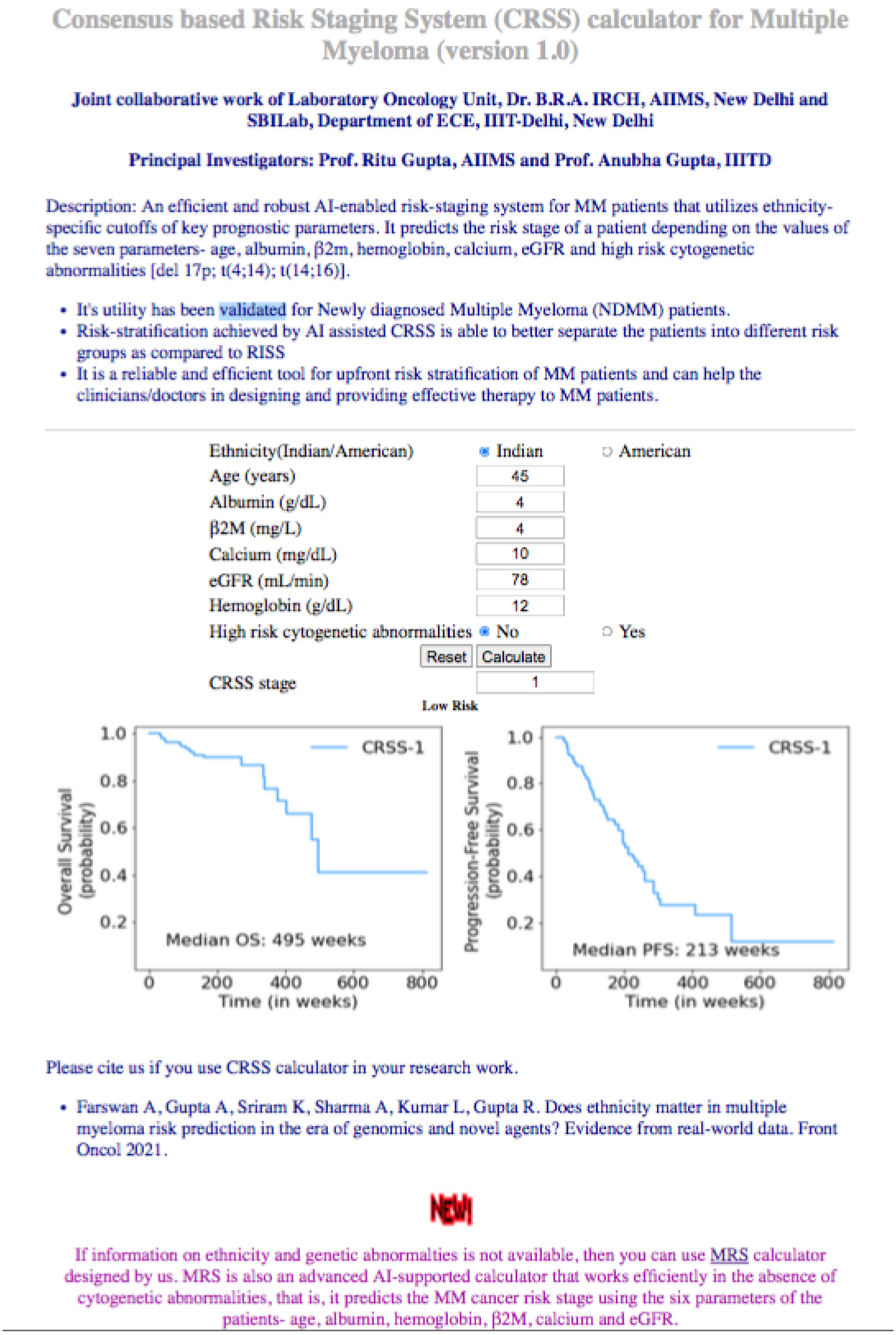

Consensus based Risk Staging System (CRSS) calculator for Multiple Myeloma (version 1.0)

Joint collaborative work of Laboratory Oncology Unit, Dr. B.R.A. IRCH, AIIMS, New Delhi and SBILab, Department of ECE, IIIT-Delhi, New Delhi

Principal Investigators: Prof. Ritu Gupta, AIIMS and Prof. Anubha Gupta, IIITD

Description: An efficient and robust AI-enabled risk-staging system for MM patients that utilizes ethnicity-specific cutoffs of key prognostic parameters. It predicts the risk stage of a patient depending on the values of the seven parameters- age, albumin, β2m, hemoglobin, calcium, eGFR and high risk cytogenetic abnormalities [del 17p; t(4;14); t(14;16)].

- It's utility has been validated for Newly diagnosed Multiple Myeloma (NDMM) patients.
- Risk-stratification achieved by AI assisted CRSS is able to better separate the patients into different risk groups as compared to RISS
- It is a reliable and efficient tool for upfront risk stratification of MM patients and can help the clinicians/doctors in designing and providing effective therapy to MM patients.

Ethnicity(Indian/American)	Indian (selected)	American
Age (years)	45	
Albumin (g/dL)	4	
β2M (mg/L)	4	
Calcium (mg/dL)	10	
eGFR (mL/min)	78	
Hemoglobin (g/dL)	12	
High risk cytogenetic abnormalities	No (selected)	Yes
Reset	Calculate	
CRSS stage	1	

Low Risk

Please cite us if you use CRSS calculator in your research work.

- Farswan A, Gupta A, Sriram K, Sharma A, Kumar L, Gupta R. Does ethnicity matter in multiple myeloma risk prediction in the era of genomics and novel agents? Evidence from real-world data. Front Oncol 2021.

NEW!

If information on ethnicity and genetic abnormalties is not available, then you can use MRS calculator designed by us. MRS is also an advanced AI-supported calculator that works efficiently in the absence of cytogenetic abnormalities, that is, it predicts the MM cancer risk stage using the six parameters of the patients- age, albumin, hemoglobin, β2M, calcium and eGFR.

Figure S7: Online version of CRSS calculator
